# Defining Potentially Unprofessional Behavior on Social Media for Health Care Professionals: Mixed Methods Study

**DOI:** 10.2196/35585

**Published:** 2022-08-09

**Authors:** Tea Vukušić Rukavina, Lovela Machala Poplašen, Marjeta Majer, Danko Relić, Joško Viskić, Marko Marelić

**Affiliations:** 1 Andrija Štampar School of Public Health School of Medicine University of Zagreb Zagreb Croatia; 2 Department of Fixed Prosthodontics School of Dental Medicine University of Zagreb Zagreb Croatia

**Keywords:** professionalism, e-professionalism, internet, social media, social networking, medicine, dental medicine, health care professionals, students, faculty

## Abstract

**Background:**

Social media presence among health care professionals is ubiquitous and largely beneficial for their personal and professional lives. New standards are forming in the context of e-professionalism, which are loosening the predefined older and offline terms. With these benefits also come dangers, with exposure to evaluation on all levels from peers, superiors, and the public, as witnessed in the #medbikini movement.

**Objective:**

The objectives of this study were to develop an improved coding scheme (SMePROF coding scheme) for the assessment of unprofessional behavior on Facebook of medical or dental students and faculty, compare reliability between coding schemes used in previous research and SMePROF coding scheme, compare gender-based differences for the assessment of the professional content on Facebook, validate the SMePROF coding scheme, and assess the level of and to characterize web-based professionalism on publicly available Facebook profiles of medical or dental students and faculty.

**Methods:**

A search was performed via a new Facebook account using a systematic probabilistic sample of students and faculty in the University of Zagreb School of Medicine and School of Dental Medicine. Each profile was subsequently assessed with regard to professionalism based on previously published criteria and compared using the SMePROF coding scheme developed for this study.

**Results:**

Intercoder reliability increased when the SMePROF coding scheme was used for the comparison of gender-based coding results. Results showed an increase in the gender-based agreement of the final codes for the category professionalism, from 85% in the first phase to 96.2% in the second phase. Final results of the second phase showed that there was almost no difference between female and male coders for coding potentially unprofessional content for students (7/240, 2.9% vs 5/203, 2.5%) or for coding unprofessional content for students (11/240, 4.6% vs 11/203, 5.4%). Comparison of definitive results between the first and second phases indicated an understanding of web-based professionalism, with unprofessional content being very low, both for students (9/222, 4.1% vs 12/206, 5.8%) and faculty (1/25, 4% vs 0/23, 0%). For assessment of the potentially unprofessional content, we observed a 4-fold decrease, using the SMePROF rubric, for students (26/222, 11.7% to 6/206, 2.9%) and a 5-fold decrease for faculty (6/25, 24% to 1/23, 4%).

**Conclusions:**

SMePROF coding scheme for assessing professionalism of health-care professionals on Facebook is a validated and more objective instrument. This research emphasizes the role that context plays in the perception of unprofessional and potentially unprofessional content and provides insight into the existence of different sets of rules for web-based and offline interaction that marks behavior as unprofessional. The level of e-professionalism on Facebook profiles of medical or dental students and faculty available for public viewing has shown a high level of understanding of e-professionalism.

## Introduction

### Background

Social media (SM) use has long become mainstream, and both our private and professional lives are daily influenced by events, changes, and developments occurring on these web-based services. Private and professional life is interchanging, and navigating this can pose a challenge, especially for health care professionals (HCPs). New standards are forming, which are possibly loosening older and predefined terms.

Professionalism is broadly defined as behavior in accordance with professional and ethical standards of the profession and can be evaluated through ten components: professional competence, honesty in a physician-patient relationship, health professional–patient privacy, maintaining a proper relationship with the patient, improving the quality of health care, improving the availability of health care, fair distribution of resources, evidence-based knowledge, maintaining patient confidence (prevention of conflict of interest), and professional responsibility [1].

e-Professionalism is a form of professionalism that can be defined as the implementation of traditional principles of professionalism during web-based activities. It is a commitment to carry out professional tasks while adhering to ethical principles and care for the patient’s well-being while using SM [2]. Cain et al [3] were the first authors who defined e-professionalism in a more concise way that can ease the operationalization of the concept as “attitudes and behaviors [some of which may occur in private settings] reflecting traditional professionalism paradigms that are manifested through digital media.” When using sociological approach, through terms of norms and sanctions that define socialization in the medical profession [4], even though e-professionalism is traditionally defined as both *attitude* and *behavior*, the behavioral part of e-professionalism is more of a concern to the medical profession, because it represents a violation of the professional norms and can be susceptible to sanctions. Other terms used in the literature for the intersection between medical professionalism and SM are online professionalism or digital professionalism [5].

A large number of medical and educational institutions [6-10] have implemented guidelines for e-professional behavior. This effort to implement, teach, and adhere to e-professional behavior emphasizes how important this concept is to the medical profession.

HCPs are increasingly encountering board disciplinary proceedings, monetary fines, and even license restrictions and suspensions due to heightened awareness of rigorous ethical and legal boundaries for web-based professional behavior [11,12]. This has also been influenced by the positive shift in patient’s attitudes toward educating themselves about their health on the web and gathering information about their physicians [12]. In addition, a new problem arises, as web-based actions and events are no longer temporary. The digital footprint is everlasting and unprofessional activity can re-emerge from past events and remains inerasable [13].

Even though research about HCPs’ professionalism issues on SM and social networking sites (SNSs) began in 2010 [14,15], researchers still name a gray area between clearly professional content and unprofessional content with various terms as questionable content [14,16], potentially or questionably unprofessional content [17-19], or potentially objectionable content [20,21]. In addition to the lack of consistent terminology, there is also no consensus on the criteria needed to define or explain what these terms constitute, nor has a validated instrument been developed to assess those types of content on SM or SNSs of HCPs.

In December 2019, a paper by Hardouin et al [22] was published in the *Journal of Vascular Surgery* investigating publicly available Facebook (FB), Twitter, and Instagram profiles of young vascular surgeons for unprofessional posts. The study screened SM profiles for prespecified material categories as either clearly unprofessional or potentially unprofessional, which was based on previously published studies of unprofessional SM content among general surgery and urology residents [17,20]. A total of 3 male researchers created new anonymous SM profiles and screened the publicly available content of the SM profiles. Clearly unprofessional content was defined as *Health Insurance Portability and Accountability Act* (HIPAA) violations, intoxicated appearance, unlawful behavior, possession of drugs or drug paraphernalia, and uncensored profanity or offensive comments about colleagues or patients. Potentially unprofessional content was defined as holding or consuming alcohol, inappropriate attire, censored profanity, controversial political or religious comments, and controversial social topics. Examples of inappropriate attire cited in the publication were provocative Halloween costumes and provocative posing in bikinis or swimwear [22].

This sparked controversy, primarily on Twitter, and the #medbikini movement started on July 23, 2020, with the tweet by Dr Londyn Robinson: “Article says photos of vascular surgeons in a ‘provocative pose wearing a bikini’ is unprofessional. I’ll say it: I wear bikinis. I am going to be a doctor. I also have a belly button ring. I am a professional person” [23]. This carried over to other SM sites and mainstream media, which criticized the lack of objectivity and bias of researchers, reviewers, and editors and created the hashtag #medbikini for the movement [24]. A great number of HCPs participated in the outrage against branding posting of such images or videos in bikinis as a possible sign of unprofessional behavior. As a revolt, they posted exactly such content with the #medbikini, showing their disapproval of such a label and referring to the gender bias of the researchers, questioning possibly outdated norms of behavior for HCPs [25]. In a month after Dr Robinson posted the original tweet [23], by the end of August 2020, the #medbikini movement gained >55,000 tweets with 40,000 contributors (Multimedia Appendix 1). Screenshots of publicly available SM reactions to #medbikini movement are presented in Multimedia Appendix 2 [23,26-30].

This has ultimately led to the retraction of the paper, invited commentary [31], and the publication of a retraction notice by the editors of the *Journal of Vascular Surgery* [22,32]. Official notice from the journal [32] was very methodologically oriented, stating the reasons for the retraction: “study did not have permission to use the list of vascular trainees, the methodology, analysis and conclusions of this article were based on published but not validated criteria, the study had significant conscious and unconscious biases caused by predominantly male authorship that supervised the assessments made by junior, male students and trainees.”

Until January 16, 2022, the #medbikini movement had reached 60,002 tweets with 40,863 contributors (Multimedia Appendix 1), 27,911 posts on Instagram, and >10,000 posts on FB (Multimedia Appendix 2). The attention that the #medbikini movement gained on SM, with numbers of an engaged audience, emphasizes the importance of e-professionalism of HCPs. It is important to raise awareness about ways that e-professionalism affects the digital footprint of all HCPs and investigate the difference between the unquestionably unprofessional posted content and very questionable potentially unprofessional content. When the #medbikini movement erupted, prior preconceptions of professionalism have started to be considered as outdated or are criticized and have potentially become dismissed. Initiatives for a new definition or at least a better understanding of the term began. In the same journal, the *Journal of Vascular Surgery*, a year after the paper by Hardouin et al [22] was published and retracted [32], Drudi et al [33] gave a historical overview of professionalism in surgery in an attempt to present a new general direction for the definition of the term professionalism. They suggested a much more inclusive definition based on diversity and equity, with responsibilities toward professionalism explained on the level of the individual, the organization, and society at large. HIPAA violations and legal transgressions remain in the realm of unprofessional behavior on SM; however, individual rights to self-expression and self-realization are loosely given priority over professionalism.

As a part of a long-term research project funded by the Croatian Science Foundation *Dangers and benefits of social networks: E-Professionalism of healthcare professionals – SMePROF* [34], the female authors of this paper engaged in content analysis of FB profiles of students and faculty of medical and dental schools in April 2020, before the #medbikini movement started.

### Objectives

The primary objective of the study was to compare professionalism on FB of medical or dental students and faculty of 2 schools in Croatia with previous research, using the rubric for assessment of unprofessional content on FB as described in the papers by Koo et al [20,21].

When the #medbikini movement happened, the gender of the coders was brought up as one of the main reasons for the bias of the study, because all the coders were young men. Besides the gender of the coders, the methodology was questionable, emphasizing that the analysis and conclusions of the study were based on published but not validated criteria. So the question raised was, how and to what extent could the imprecisely defined or explained subcategories of potentially unprofessional content in the studies by Langenfeld et al [17,18] and Koo et al [20,21] be accounted for the #medbikini reaction, as they were used as a basis for the coding criteria in the retracted paper by Hardouin et al [22].

With the unique position of having an objective, nonbiased data from the pre-#medbikini era and from female coders, we decided to extend and enhance the primary objective and to set new objectives for a more complex study. Objectives of this study were to (1) develop an improved coding scheme (SMePROF coding scheme) for the assessment of unprofessional behavior on FB of medical or dental students and faculty, (2) compare reliability between coding schemes used in previous research and SMePROF coding schemes, (3) compare gender-based differences for the assessment of the professional content on FB, (4) validate the SMePROF coding scheme, and (5) assess the level of and to characterize web-based professionalism on publicly available FB profiles of medical or dental students and faculty.

## Methods

### Chronology and Research Design

**Figure 1:**
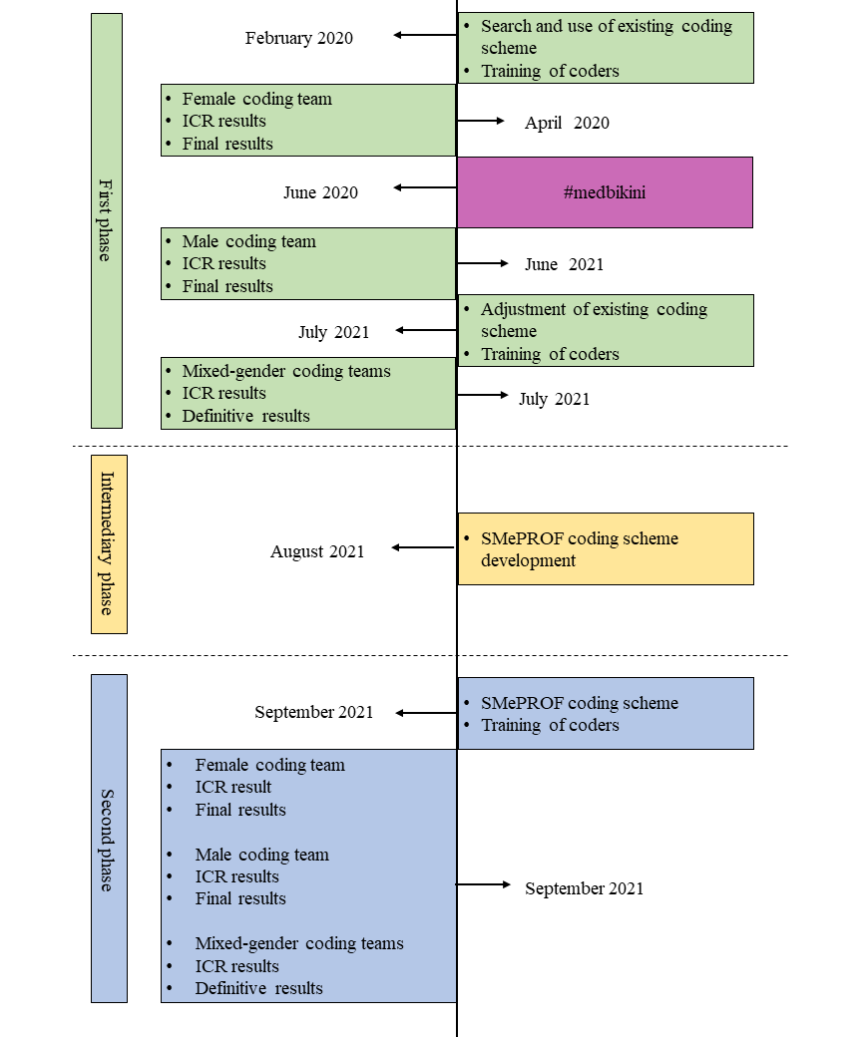
Phases of the study. ICR: intercoder reliability.

The study was conducted in 3 phases, the first phase, the intermediary phase, and the second phase (Figure 1). The first phase included three rounds of coding: female coding team, male coding team, and mixed-gender coding team, using the coding scheme for assessment of unprofessional behavior on FB, developed for this study based on previous research (Nason-Koo coding scheme), presented in Multimedia Appendix 3 [16,20,21]. After the initial part of the first phase (female coding), the #medbikini movement occurred which influenced the rest of the research design.

The intermediate phase was the development of the new rubric for assessment of unprofessional content on FB (SMePROF rubric), which included corrections of the rubric for assessment of unprofessional content (Koo rubric) by Koo et al [20,21] based on findings from the previous steps and insights from the #medbikini movement [22,31,32,35-38], resulting in the new SMePROF coding scheme, especially in the category for assessment of unprofessional content (SMePROF rubric). Finally, the second phase included three rounds of coding: female coding team, male coding team, and mixed-gender coding team, using the SMePROF coding scheme that includes the SMePROF rubric.

### Ethics Approval

Following approval from the ethical committees of the School of Medicine University of Zagreb (UZSM) and the School of Dental Medicine University of Zagreb (UZSDM), UZSM (641-01/18-02/01) and UZSDM (05-PA-24-2/2018), students’ class lists were obtained from the schools’ secretarial staff for all years. Lists of all faculty were also obtained for both schools. Authors were blinded to the individual student identification numbers.

To view individual student or faculty FB profiles, coders used a neutral, newly created FB account in each coding round. This account was intended to mimic potential search queries from patients, employers, or members of the public and capture publicly available content. Because these neutral accounts had no connections to other accounts of the inspected profiles, it was ensured that the content considered was accessible to any member of the public. The only data that were analyzed was the information that students or faculty made publicly available; hence, the posts that anyone, regardless of the friendship status with them on FB, could see were analyzed. The final samples of UZSM and UZSDM students and faculty were searched on FB, individually by name from the lists, by each author using the newly created FB account.

### Instruments Used in the Study

In the first phase of the study, profiles were reviewed for 6 categories according to the coding scheme developed for this study based on previous research (Nason-Koo coding scheme) [16,20,21]. The Nason-Koo coding scheme consists of six categories previously used in the study by Nason et al [16]: (1) existence of identifiable FB profile, (2) sex, (3) privacy settings, (4) relationship status revealed, (5) affiliation with the school revealed, and (6) professionalism. Category professionalism was coded according to the rubric for assessment of unprofessional content on Facebook (Koo rubric) by Koo et al [20,21]. The Nason-Koo coding scheme and Koo rubric are presented in Multimedia Appendix 3.

Because one of the objectives of the study is to explore how and to what extent could the imprecisely defined or explained subcategories of potentially unprofessional behavior in previous studies [17-21] be accounted for the #medbikini reaction, as they were used as a basis for the coding criteria in the retracted paper by Hardouin et al [22], we have developed a SMePROF rubric for assessment of unprofessional content on FB during the intermediate phase of the study (Figure 1). Changes were made during August 2021, resulting in a SMePROF coding scheme that differs from the Nason-Koo coding scheme based on the changes made in the SMePROF rubric (Textbox 1).

**SMePROF rubric for assessment of unprofessional content on Facebook.**

**Unprofessional content**

- Image
  Protected health information
  Engaging in unlawful behavior
  Offensive attire (photo or video content of an attire that includes offensive elements; for example, wearing a T-shirt with profanity or Nazi symbols [work or nonwork related])
  Possession of drugs or appearance thereof
  Displaying drug paraphernalia
  Appearing intoxicated
  Offensive content of a political, religious, or racial nature
- Text
  Protected health information
  References to specific instances of unlawful behavior
  References to possession of drugs
  References to drug paraphernalia
  References to specific instances of alcohol intoxication
  Uncensored profanity
  Offensive comments about colleagues at own hospitals
  Offensive comments about colleagues at other hospitals
  Offensive comments about a specific patient
  Offensive comments of a political, religious, or racial nature
- Page, link, or other posted content
  Advocating or supporting the use of drugs
  Advocating or supporting unlawful behavior
  Offensive content of a political, religious, or racial nature

**Potentially unprofessional content**

- Image
  Holding or consuming alcohol in a clinical or work-related setting (excluding conferences or other work-related dinners or parties)
  Inappropriate attire (clinical or work-related setting: photo or video content in a clinical or work environment in which an individual is wearing physicians’ attire [laboratory coat, scrubs, surgical gowns, etc] and also partially revealing skin [sleeveless, deep cleavage, abdomen, back, short pants, or skirts high above the knee] or underwear inappropriate for clinical or work environment)
  Sexualization—sexually suggestive or provocative posing regardless of the attire or revealing clothing (sexualization focuses on sexual suggestive or provocative posing [in a professional or private setting], regardless of the attire or revealing clothing, excluding nonsexual suggestive posing in swim or beachwear)
- Text
  Reference to sexually provocative or sexually disturbing content
  Censored profanity
- Page, link, or other posted content
  Advocating or supporting alcohol intoxication
  Sexually provocative or sexually disturbing content

Main differences comparing the Koo rubric and the SMePROF rubric for assessment of unprofessional content on FB are presented in Multimedia Appendix 4 [17,18,20-22,33,36,37, 39,40].

In the second phase of the study, profiles were reviewed for six categories (existence of identifiable FB profile, sex, privacy settings, relationship status revealed, affiliation with the school revealed, and professionalism) according to SMePROF coding scheme. Category professionalism was coded according to the SMePROF rubric. Profiles were categorized as (1) unprofessional content if at least one element of unprofessional content was found, (2) potentially unprofessional content if at least one element of potentially unprofessional content was found or, (3) professional content if none of the elements of unprofessional nor potentially unprofessional content were found (Textbox 1).

### Coders and Coding Process

In each step of the coding processes, assessments were first conducted by the two independent coders (either members of female or male coding teams). The female coders were LMP and M Majer. LMP, aged 37 years, was at that time a Master of Library and Information Science; M Majer, aged 45 years, was at that time a doctor of medicine (MD), a school and adolescent medicine specialist, and an assistant professor in public health. The male coders were DR and JV. DR, aged 33 years, was at that time an MD and a family medicine resident, and JV, aged 36 years, was at that time a doctor of dental medicine (DMD), a prosthodontics specialist, and an assistant professor of dental medicine.

Intercoder reliability (ICR) was determined for subjective category professionalism for female and male coding teams in both phases of the study. ICR was determined using the following indices: *Average Pair-Wise Percent Agreement* and *Krippendorff* α [41]. For differences between 2 coders, the first consensus among coders was tried to be established; if not able to reach a consensus, a third reviewer was consulted and differences were resolved (the third reviewer, TVR, was always the same: woman; aged 46 years; an MD, a psychiatrist, and an assistant professor in public health). This process produced the final results for the female and male coding teams in the first and second phases of coding (Figure 1).

The final results of the female and male coders from the first and second phases of coding were compared, and the ICR was determined for the categories as in the previous steps. If there were differences in the final results between the 2 teams, an attempt was first made to reach a consensus between the teams. If this was not possible, a third reviewer (TVR) was consulted, and the differences were resolved. This led to definitive results produced for both genders in both phases of coding (Figure 1).

### Statistical Analysis

ICR during both phases of coding was determined for the subjective category professionalism using the indices Average Pair-Wise Percent Agreement and Krippendorff α [41]. ICR was calculated with the ReCal (*Reliability Calculator*), an online utility that computes ICR coefficients [42].

Descriptive statistics were used to present all data obtained in both phases. Differences between coders’ variable categories within the same coding team and phase were assessed using the chi-square test or Fisher test, if >20% of cells had an expected count of <five. Differences among ordinal variables conducted between the first and the second phases were tested using the Wilcoxon signed-rank test. *P* values of <.05 were considered statistically significant. All statistical analyses were carried out using the SPSS Statistics (version 26; IBM) software.

## Results

### Overview

The final samples for the content analysis of student’s and faculty’s FB profiles were made by a method of systematic sampling, therefore allowing us to create probabilistic samples that represent populations better than random sampling [43]. The sample of students’ FB profiles included 16.7% of all registered students at the UZSM (325/1951) and 16.6% of students at the UZSDM (94/566), equally distributed according to study year and gender (n=419). The final sample for the content analysis of faculties’ FB profiles was made by systematic sampling of 16.7% of all registered faculty at the UZSM (86/516) and 16.9% at the UZSDM (28/166), equally distributed according to the academic position and gender(n=114). In total, there were 533 names for analysis (n=419, 79% students and n=114, 21% faculty).

In the first phase of coding, the female coding team found 255 (60.9%) students and 42 (36.8%) faculty with identifiable FB accounts. The male coding team found 222 (53%) students and 24 (21.1%) faculty with identifiable FB accounts. Definitive results (mixed-gender coding team) for the first phase of coding found 222 (53%) students and 25 (21.9%) faculty with identifiable FB accounts (*χ*^2^_2_=63.7; *P*<.001). In the second phase of coding, the female coding team found 240 (57.3%) students and 41 (36%) faculty with identifiable FB accounts. The male coding team found 203 (48.4%) students and 24 (21.1%) faculty with identifiable FB accounts. Definitive results (mixed-gender coding team) for the second phase of coding found 206 (49.2%) students and 23 (20.2%) faculty with identifiable FB accounts (*χ*^2^_2_=30.7; *P*<.001).

### ICR Results

ICR results for female coding team, male coding team, and final female versus final male coding teams in the category professionalism, for the first (Koo rubric) and the second (SMePROF rubric) phases are presented in Table 1.

ICR shows an increase when the SMePROF rubric was used for gender-based coding and for comparison of final results.

**Table 1:** Intercoder reliability for the total sample.

	Category (professionalism)				
	Koo rubric			SMePROF rubric	
	APPA^a^	Krippendorff α	APPA		Krippendorff α
Female coding team	79.40	.64	81.10		.71
Male coding team	79.20	.61	82.40		.67
Final female versus final male coding teams	76.90	.60	82.00		.67

^a^APPA: Average Pair-wise Percent Agreement.

### Comparison of the Gender-Based Differences Among Coder Teams for the Category Professionalism (First and Second Phases of Coding)

Comparison of the gender-based difference among coder teams for the category professionalism according to the Koo and SMePROF rubric is presented in Table 2.

Final results show that while using the Koo rubric, the female coding team, more often than the male coding team, reported potentially unprofessional content (54/297, 18.2% vs 29/246, 11.8%) but almost 2.5 times less than the male coding team, reported unprofessional content (5/297, 1.7% vs 10/246, 4.1%).

Final results show that in the second phase (SMePROF rubric), there was almost no difference between female and male coding teams for coding potentially unprofessional content (8/281, 2.9% vs 6/227, 2.6%) or for coding unprofessional content (13/281, 4.6% vs 11/227, 4.9%).

When we compared the final female coding with the final male coding (for accounts that both teams coded as identifiable FB accounts, n=227), the gender-based agreement using the Koo rubric was 85% for the reviewed profiles (Table 3). In the second phase of coding, for the comparison of final female coding and final male coding (for accounts that both teams coded as identifiable FB accounts, n=210), gender-based agreement using the SMePROF rubric was 96.2% for the profiles reviewed (Table 3).

**Table 2:** Gender-based differences for the category professionalism (Koo rubric vs SMePROF rubric).

Difference	Koo rubric			SMePROF rubric (N=508)	
	Female (n=297), n (%)	Male (n=246), n (%)	Female (n=281), n (%)		Male (n=227), n (%)
None	238 (80.1)	207 (84.1)	260 (92.5)		210 (92.5)
Potentially unprofessional	54 (18.2)	29 (11.8)	8 (2.9)		6 (2.6)
Unprofessional	5 (1.7)	10 (4.1)	13 (4.6)		11 (4.9)

**Table 3:** Gender-based agreement of the final codes for the category professionalism (Koo rubric vs SMePROF rubric).

Agreement	Comparison (Koo rubric) of final female coding with final male coding (n=227), n (%)	Comparison (SMePROF rubric) of final female coding with final male coding (n=210), n (%)
Unprofessional↔none	1 (0.4)	2 (1)
Unprofessional↔potentially unprofessional	4 (1.8)	4 (1.9)
Unprofessional↔unprofessional	4 (1.8)	9 (4.3)
Potentially unprofessional↔potentially unprofessional	17 (7.5)	4 (1.9)
Potentially unprofessional↔none	29 (12.8)	2 (1)
None↔none	172 (75.8)	189 (90)
Subtotal disagreement	34 (15)	8 (3.8)
Subtotal agreement	193 (85)	202 (96.2)

### Comparison of Definitive Results Between the First and the Second Phases

#### Comparison of the Koo and SMePROF Rubric Results for the Category Professionalism, Female Coding Versus Male Coding Versus Definitive Coding

Table 4 displays a comparison of the definitive results for the category professionalism (Koo vs SMePROF rubric), divided between students and faculty, for the total sample (N=533).

**Table 4:** Comparison of the Koo and SMePROF rubric coding results for the category professionalism, final female coding versus final male coding versus definitive coding (N=533).

Group and professionalism			Final female coding				Final male coding					Definitive coding		
			Koo rubric, n (%)	SMePROF rubric, n (%)		Koo rubric, n (%)			SMePROF rubric, n (%)		Koo rubric, n (%)			SMePROF rubric, n (%)
**Students (n=419)**														
	No unprofessional content	208 (81.6)		222 (92.5)	188 (84.7)			187 (91.7)		187 (84.2)			188 (91.3)	
	Potentially unprofessional content	43 (16.9)		7 (2.9)	25 (11.3)			5 (2.5)		26 (11.7)			6 (2.9)	
	Unprofessional content	4 (1.6)		11 (4.6)	9 (4.1)			11 (5.4)		9 (4.1)			12 (5.8)	
	No profile or impossible to determine	164 (39.1)		179 (42.7)	197 (47)			216 (51.6)		197 (47)			213 (50.8)	
**Faculty (n=114)**														
	No unprofessional content	30 (71.4)		38 (92.7)	19 (79.2)			23 (95.8)		18 (72)			22 (95.7)	
	Potentially unprofessional content	11 (26.2)		1 (2.4)	4 (16.7)			1 (4.2)		6 (24)			1 (4.3)	
	Unprofessional content	1 (2.4)		2 (4.9)	1 (4.2)			0 (0)		1 (4)			0 (0)	
	No profile or impossible to determine	72 (63.2)		74 (64.9)	90 (78.9)			90 (78.9)		89 (78.1)			91 (79)	

In a sample of students, a comparison of the Koo and SMePROF rubric results showed a decrease in potentially unprofessional content for final female coding (from 43/255, 16.9% to 7/240, 2.9%), final male coding (from 25/222, 11.3% to 5/203, 2.5%), and definitive coding (from 26/222, 11.7% to 6/206, 2.9%). Decrease in potentially unprofessional content was also observed in the sample of faculty for final female coding (from 11/42, 26.2% to 1/41, 2.4%), final male coding (from 4/24, 17% to 1/24, 4%), and definitive coding (from 6/25, 24% to 1/23, 4%). On the contrary, when comparing students’ sample Koo and SMePROF rubric results, an increase in unprofessional content was shown for final female coding (from 4/255, 1.6% to 11/240, 4.6%), final male coding (from 9/222, 4.1% to 11/203, 5.4%), and definitive results (from 9/222, 4.1% to 12/206, 5.8%).

Similar decrease in potentially unprofessional content was observed in the faculty’s sample for final female coding (from 11/42, 26% to 1/41, 2%), final male coding (from 4/24, 17% to 1/24, 4%), and for definitive coding (from 6/25, 24% to 1/23, 4%). When comparing the faculty’s sample Koo and SMePROF rubric results, an increase in unprofessional content was shown only for final female coding (from 1/42, 2% to 2/41, 5%), but final male coding (from 1/24, 4% to 0/24, 0%) and definitive coding (from 1/25, 4% to 0/23, 0%) showed a decrease in unprofessional content.

#### Unprofessional or Potentially Unprofessional Content on Students’ and Faculty’s Public Facebook Accounts

The categories and frequencies of unprofessional or potentially unprofessional content using the Koo rubric are summarized in Table 5.

The categories and frequencies of unprofessional or potentially unprofessional content using the SMePROF rubric are summarized in Table 6.

**Table 5:** Unprofessional or potentially unprofessional content on the students’ and faculty’s public Facebook accounts (Koo rubric).

Content type and content				Final female coding			Final male coding					Definitive coding		
				Students (n=255), n (%)	Faculty (n=42), n (%)		Students (n=222), n (%)		Faculty (n=24), n (%)		Students (n=222), n (%)			Faculty (n=25), n (%)
**Unprofessional content**				4 (1.6)	1 (2.4)		9 (4.1)		1 (4.2)		9 (4.1)			1 (4)
	Uncensored profanity (T^a^)			1 (0.4)	0 (0)		4 (1.8)		0 (0)		5 (2.3)			0 (0)
	Appearing intoxicated (I^b^)			1 (0.4)	0 (0)		1 (0.5)		0 (0)		2 (0.9)			0 (0)
	Advocating or supporting the use of drugs (P^c^)			1 (0.4)	0 (0)		2 (0.9)		0 (0)		1 (0.5)			0 (0)
	Protected health information (I or T)			0 (0)	0 (0)		1 (0.5)		0 (0)		1 (0.5)			0 (0)
	Offensive attire (I)			1 (0.4)	1 (2.4)		0 (0)		0 (0)		0 (0)			0 (0)
	References to alcohol intoxication (T)			0 (0)	0 (0)		1 (0.5)		0 (0)		1 (0.5)			1 (4)
**Potentially unprofessional content**				43 (16.9)	11 (26.2)		25 (11.3)		4 (16.7)		26 (11.7)			6 (24)
		Holding alcohol (I)	8 (3.1)		2 (4.8)	5 (2.3)		1 (4.2)		8 (3.6)			0 (0)	
		Appearing in sexually suggestive attire or circumstances (I)	13 (5.1)		1 (2.4)	3 (1.4)		1 (4.2)		6 (2.7)			2 (8)	
		Inappropriate attire (I)	13 (5.1)		3 (7.1)	7 (3.2)		1 (4.2)		5 (2.3)			0 (0)	
		Censored profanity (T)	2 (0.8)		0 (0)	4 (1.8)		0 (0)		2 (0.9)			0 (0)	
		References to sex or sexual behavior (T)	2 (0.8)		1 (2.4)	1 (0.5)		0 (0)		1 (0.5)			0 (0)	
		References to alcohol intoxication (T)	2 (0.8)		0 (0)	1 (0.5)		0 (0)		1 (0.5)			0 (0)	
		Politics or content of a political nature (P)	1 (0.4)		2 (4.8)	1 (0.5)		1 (4.2)		1 (0.5)			1 (4)	
		Consuming alcohol (I)	2 (0.8)		1 (2.4)	3 (1.4)		0 (0)		1 (0.5)			3 (12)	
		Controversial of polarizing topic (P)	0 (0)		1 (2.4)	0 (0)		0 (0)		0 (0)			0 (0)	

^a^T: text.

^b^I: image.

^c^P: post, link, or other posted content.

**Table 6:** Unprofessional or potentially unprofessional content on the students’ and faculty’s public Facebook accounts (SMePROF rubric).

Content type and content		Final female coding		Final male coding		Definitive coding	
		Students (n=240), n (%)	Faculty (n=41), n (%)	Students (n=203), n (%)	Faculty (n=24), n (%)	Students (n=206), n (%)	Faculty (n=23), n (%)
**Unprofessional content**		11 (4.6)	2 (4.9)	11 (5.4)	0 (0)	12 (5.8)	0 (0)
	Possession of drugs or appearance thereof (I^a^)	2 (0.8)	0 (0)	0 (0)	0 (0)	0 (0)	0 (0)
	Appearing intoxicated (I)	2 (0.8)	0 (0)	2 (1.0)	0 (0)	2 (1)	0 (0)
	Uncensored profanity (I or T^b^)	5 (2.1)	1 (2.4)	5 (2.5)	0 (0)	5 (2.4)	0 (0)
	Offensive attire (I)	2 (0.8)	0 (0)	1 (0.5)	0 (0)	2 (1)	0 (0)
	Offensive comments of a political, religious, or racial nature (T)	0 (0)	1 (2.4)	0 (0)	0 (0)	0 (0)	0 (0)
	Advocating or supporting the use of drugs (T or P^c^)	0 (0)	0 (0)	1 (0.5)	0 (0)	1 (0.5)	0 (0.0)
	Offensive content of a political, religious, or racial nature (T or I)	0 (0)	0 (0)	2 (1.0)	0 (0)	2 (1.0)	0 (0)
**Potentially unprofessional content**		7 (2.9)	1 (2.4)	5 (2.5)	1 (4)	6 (2.9)	1 (4)
	Inappropriate or offensive attire (nonsexual; I)	1 (0.4)	0 (0)	1 (0.5)	0 (0)	0 (0)	0 (0)
	Sexualization—appearing in sexually suggestive posture (I)	3 (1.3)	1 (2.4)	3 (1.5)	1 (4)	4 (1.9)	1 (4)
	Advocating or supporting alcohol intoxication (T or P)	1 (0.4)	0 (0)	1 (0.5)	0 (0.0)	2 (1)	0 (0)
	Sexually provocative or sexually disturbing content (I)	2 (0.8)	0 (0)	0 (0)	0 (0)	0 (0)	0 (0)

^a^I: image.

^b^T: text.

^c^P: post, link, or other posted content.

### Definitive Results

For the definitive results, significant differences between students and faculty were identified regarding the existence of identifiable FB accounts (206/419, 49.2% vs 23/114, 20.2%; *χ*^2^_1_=30.7; *P*<.001), affiliation of the school revealed (193/206, 93.7% vs 15/23, 65%; *χ*^2^_1_=20.1; *P*<.001), and relationship status revealed (11/206, 5.3% vs 4/23, 17%; *χ*^2^_1_=4.9; *P*=.03). There were no significant differences between students and faculty for closed privacy settings (202/206, 98.1% vs 21/23, 91%; *χ*^2^_2_=3.7; *P*=.11) or for the category professionalism (*χ*^2^_2_=1.5; *P*=.47). Although there were no statistically significant differences between students and faculty in definitive results of professionalism variable, students had less potentially unprofessional content than faculty (6/206, 2.9% vs 1/23, 4%); however, they had more unprofessional content (12/206, 5.8% vs 0/23, 0%).

## Discussion

### Comparison With Previous Research

The consensus about what constitutes unprofessional behavior has still not been reached since the original definition by Chretien et al [15] in 2010. There are numerous studies with examples of definitions of unprofessional behavior on SM [44-48].

From the first study in 2013 by Ponce et al [14] to the latest study in 2021 by Pronk et al [49], various attempts were made to define the gray area of e-professionalism. This has brought a great deal of variety to the field, possibly even causing one of the biggest medical affairs on SM—#medbikini. Understanding the evolution and difficulty of defining this problem, defining the linguistic terms and nuances, and the ramifications of ill-fated attempts to do so are crucial to our research; therefore, a review of previous research descriptions of potentially unprofessional content is provided in Multimedia Appendix 5 [6,14,17,18,20-22,49-52].

The #medbikini movement has raised important questions, besides professional questionability of posting pictures in bikinis for our female colleagues, also regarding the possibility to present ourselves as humans on SM or to be able to express an opinion about important social topics. HCPs have realized that SM is not just a platform to post vacation photos and interact with followers [53]. As Drudi et al [33] emphasized, there is a need to re-examine current definitions and philosophies surrounding professionalism in medicine that may be discriminatory and exclusive. The term professionalism has no standard definition [54]; however, resolving problems of unprofessional posting with repression is an unsustainable model of managing e-professionalism.

### Principal Findings

The #medbikini affair and the subsequent movement that followed rattled the foundations of how professional behavior is understood and valued in modern and emerging environments. With a broader understanding of the problem at hand, this paper is the first to address issues not previously reported in the literature.

We have developed the SMePROF coding scheme for the assessment of unprofessional behavior on FB by medical or dental students and faculty. The first 5 categories of the coding scheme are objective (Multimedia Appendix 3) and have remained the same in the SMePROF coding scheme, but the category professionalism has provoked many controversies so far.

Our SMePROF rubric for assessment of unprofessional content on FB was developed with the intention to improve previous instruments and rubrics, to have more precise criteria or explanations for previously ambiguous or vaguely defined categories of unprofessional or potentially unprofessional content to have fewer possibilities for subjective interpretation, and to have a more updated comprehension of e-professionalism.

The SMePROF rubric differentiates *offensive attire* versus *inappropriate attire*, inappropriate attire being defined for clinical or work-related settings. General guidance to the medical community regarding physician’s attire outside the operating room exists. The review by Bearman et al [55] showed that overall, patients express preferences for certain types of attire, with most surveys indicating a preference for formal attire, including a preference for a white coat. However, patient comfort, satisfaction, trust, and confidence in their physicians are unlikely to be affected by the practitioner’s attire choice. Petrilli et al [56] explored whether physician attire can affect patient experiences. Their findings include the fact that attire preferences vary by geographic location, patient age, and context of care. Although physician attire cannot replace excellent clinical care, data from this study suggest that it may influence how patients perceive care and perhaps how willing they are to trust their physicians. Xun et al [57] recently investigated how the public perceives casual physician attire compared with white coats and whether there are differences by gender of the physician. Their findings suggest that individuals prefer that physicians wear white coats and that gender biases in the perception of professional physician attire exist. Physician attire is only a small aspect of the practice of medicine and does not embody the wearer’s qualifications, nor does it necessarily affect their performance, practice, and contributions.

We propose a new subcategory *sexualization* in the potentially unprofessional behavior, with an explanation that sexualization focuses on sexually suggestive or provocative posing (in a professional or private setting), regardless of the attire or revealing clothing, excluding nonsexual suggestive posing in swim or beachwear. Previous researches [17,18,20-22] have not clearly defined it, which led to broad possibilities for interpretation of this category, also resulting in the #medbikini movement.

Sexualization can be envisioned as the combination of a multitude of sexualized attributes—body position, the extent of nudity, textual cues, and more—the cumulative effect of which is to narrow the possible interpretations of the image to just the sex. Sexually suggestive posture is a potentially important aspect of sexualization, because it represents open body language that appears to invite sexual activity. It can be illustrated in subtle ways such as placing a hand on one’s hips and not-so-subtle ways such as sitting with one’s legs spread wide open [58].

Although suggestive postures and revealing clothing often go hand in hand, it may be possible to decouple these elements in the media and interpersonal interactions. Bernard et al [59] have deconstructed sexualization and shown that posture suggestiveness causes objectification and exerts a more powerful influence on objectification than the skin-to-clothing ratio. For example, images of underwear and swimsuits may show people in a way that would reduce the risk of objectification by presenting them in revealing clothing but with nonsuggestive posture, so that there is no element of sexualization.

ICR results showed an increase in the Krippendorff α coefficient when the SMePROF rubric was used, both for gender-based coding (for male coders from .61 to .67; for female coders from .64 to .71) and for the comparison of final results (from .60 to .67). Although these are acceptable levels for reliability, none of these results can be classified as highly reliable [41]. Compared with the results of Ponce et al [14] for their professionalism, interrater reliability scores (Cohen κ=0.43), our results indicate a more reliable coding method. Langenfeld et al [17] were not able to calculate ICR owing to a collaborative approach and authors’ discussion of the analyzed content, but in the second paper [18], they report that κ coefficient was used to calculate interrater reliability, with no mention of the obtained results for the κ coefficient. Koo et al [20,21] in both studies reported excellent results for interreviewer concordance (κ>0.90) in all content categories. Because their description of the coding process was minimal, it is hard to speculate how these excellent interrater concordance results were obtained.

Krippendorff [41] and Potter and Levine‐Donnerstein [60] define three types of reliability: *stability, reproducibility, and accuracy* [41,60]. The first goal of this paper was to develop an improved coding scheme for the assessment of unprofessional behavior on FB, so using any of the previous instruments [17,18,20-22] as a standard for accuracy is simply impermissible and violates the purpose of this research. The stability of the instrument used in this research is on an acceptable level, because repeated measures are performed by the same coders at 2 time points and similar results are provided. However, because of the circumstances that had arisen around the #medbikini movement and necessary changes in the coding scheme, the demonstration of stability must be taken with caution. Therefore, the reliability of this instrument is mainly demonstrated through its reproducibility, with carefully selected independent coders, and further substantiated using the reliability coefficient.

This research is the first assessment of unprofessional behavior on FB comparing gender differences in the coding process itself. Besides the study by Hardouin et al [22], previous studies have not identified explicitly who among the authors were coders, according to their gender, age, academic status, or position. In the first phase of this study, coding done by the female coding team was conducted before the #medbikini movement, and it was to our knowledge the first analysis in literature done by women-only coding team before the #medbikini movement and the potential male bias emphasized as a reason for the retraction of the paper [22].

The final results of the first phase show that while using the Koo rubric, female coders more often than male coders reported potentially unprofessional content (54/297, 18.2% vs 29/246, 11.8%) but almost 2.5 times less than male coders reported unprofessional content (5/297, 1.7% vs 10/246, 4.1%). Our finding that female coders have recognized more potentially unprofessional content contradicts arguments from the retraction notice [32] and public reaction that the study by Hardouin et al had significant conscious and unconscious biases caused by predominantly male authorship that supervised the assessments made by junior male students and trainees [22,39]. Especially when our results show that results for the subcategories *inappropriate attire* or *sexually suggestive attire* final female versus final male coding (for students) were 10.2% (26/255) versus 4.5% (10/222). The lower results of unprofessional and potentially unprofessional content of male coders can be explained with a bias connected to the #medbikini movement. As the first male coding occurred after the #medbikini movement and with the knowledge (conscious or unconscious) of the new trend in understanding the boundaries of professional behavior, the results could be skewed to the lower levels. It can be argued that this also boosts the idea that there is no difference in male versus female coding as the first phase female coding results (before and unaffected by #medbikini) show a stricter approach to professionalism rooted perhaps more in outdated professionalism norms (before #medbikini) and use of an imprecise coding instrument rather than in gender differences.

During the intermediate phase and the process of the development of the SMePROF rubric, the main conclusions from coding experience in the first phase, from both female and male coders, were that coders were confused about the difference between these 2 categories and whether photographs in bikini or swimwear should be categorized as one of them. The final results of the second phase show that there was almost no difference between female and male coders for coding potentially unprofessional content for students (7/240, 2.9% vs 5/203, 2.5%) or for coding unprofessional content for students (11/240, 4.6% vs 11/203, 5.4%). Thus, we conclude that the SMePROF rubric is a more objective instrument. This is also confirmed by the increase in the gender-based agreement of the final codes for the category professionalism, from 85% (193/227) in the first phase to 96.2% (202/210) in the second phase. Gender-based differences were almost neutralized using the SMePROF rubric. A comparison of gender-based agreement of final codes in the first phase shows that majority of disagreements were detected when coders disagreed on whether the profile was potentially unprofessional or professional. This also proves that the previously defined subcategory of potentially unprofessional content [20,21] was subjective for interpretation.

The original objective of this study was to assess the level of web-based professionalism on FB profiles of medical or dental students and faculty available for public viewing. In previous studies that made a distinction between unprofessional and potentially unprofessional content [16-18,20-22], unprofessional content ranged from 2% to 12% and potentially unprofessional content, from 10.3% to 34%. Our definitive results of the first phase, using the Koo rubric, for unprofessional content (9/222, 4.1% students; 1/25, 4% faculty) are similar to the study by Nason et al [16] for students (3%) and to the study by Langenfeld et al [18] for faculty. For potentially unprofessional content, our definitive results of the first phase (26/222, 11.7% students; 6/25, 24% faculty) are lower compared with 34% of students in the study by Nason et al [16] and similar to 14.1% residents in the study by Langenfeld et al [17]. Langenfeld et al [18] determined lower rates of faculty with potentially unprofessional content (10.3%) compared with ours (6/25, 24%). The findings of Koo et al of potentially unprofessional content among urologist graduates and residents (26.9% and 25.3%, respectively) [20,21] are similar to our results for faculty (6/25, 24%).

Karveleas et al [61], in a recent study (2021) about the relationship between FB behavior and e-professionalism among Greek dental students, did not differentiate unprofessional from potentially unprofessional content. In the study, unprofessional content, defined according to previously published studies [44-47,62,63], had been posted by most participants and depicted as 71.7% posted pictures from holidays; 41.5%, moments in nightclubs; and 26.2%, photographs wearing swimwear or underwear. Still, this publication did not gain so much attention.

Comparison of definitive results between the first and second phases, indicate an understanding of web-based professionalism, with unprofessional content being very low, both for students (9/222, 4.1% to 12/206, 5.8%) and faculty (1/25, 4% to 0/23, 0%). For assessment of the potentially unprofessional content, we observed a 4-fold decrease using the SMePROF rubric for students (26/222, 11.7% to 6/206, 2.9%) and a 5-fold decrease for faculty (6/25, 24% to 1/23, 4.3%). This can be explained by a more precise definition of subcategories of the potentially unprofessional category (inappropriate attire and appearing in sexually suggestive attire or circumstances) and decreased numbers of subcategories (such as not having holding or consuming alcohol) described in the SMePROF rubric.

During the coding process of the first phase, coders themselves were confused what are the differences between inappropriate attire and appearing in sexually suggestive attire or circumstances, especially considering photographs in swimwear. Questioning whether they belong to this category or not sparked many disagreements among coders. These types of images, inappropriate attire, and appearing in sexually suggestive attire or circumstances, were observed in 5% (11/222) of the students and 8% (2/25) of the faculty, being the second most frequent potentially unprofessional content. Since we have introduced sexualization as a subcategory with a clear distinction from inappropriate attire in the SMePROF rubric, sexualization was observed for 1.94% (4/206) of the students and 4% (1/24) of the faculty. There were no examples for the subcategory inappropriate attire.

Of the profiles that we were able to access publicly in the second phase of coding (using the SMePROF rubric), although there were no statistically significant differences between students and faculty in definitive results of the professionalism variable (*χ*^2^_2_=1.5; *P*=.47), students had less potentially unprofessional content than faculty (6/206, 2.9% vs 1/23, 4%); however, they had more unprofessional content (12/206, 5.8% vs 0/23, 0%). The most frequent unprofessional content for students was uncensored profanity (5/206, 2.4%), and the faculty did not have any profile with unprofessional content. In the potentially unprofessional content, faculty had 4% (1/23) of sexualization (but this is owing to only one sexually suggestive photograph and small numbers of faculty with identifiable FB accounts, n=23). Sexualization was found in just 1.9% (4/206) of students. Images of inappropriate attire were not found within students’ or faculty profiles.

Besides more precise criteria in the SMePROF rubric, a decrease in unprofessional and potentially objectionable content in the second phase of coding could also be explained by the development of guidelines for e-professionalism for medical and dental students in UZSM and UZSDM that became publicly available in November 2020 [64]. Also, both schools have implemented in their curriculum themes e-professionalism as part of the obligatory subjects. Also, elective subjects, completely focused on e-professionalism in medicine and dental medicine have been developed and implemented in the curricula.

Although it has passed almost 20 years since the advent of SM, little evidence exists to inform about the interplay between personal web-based disclosures and professional trust and credibility from patients’ or public perspectives. Jain et al [65] measured the perception of unprofessional content of HCPs on SNSs among medical students, faculty members, and the public. The most significant result they found is that faculty members, medical students, and the public have different thresholds of what is acceptable on SM or SNS. Medical students were more likely to post comments, images, and photographs that medical school faculty members and the public would consider inappropriate or unprofessional [65]. The study by Weijs et al [66] provided the first evidence of the impact of HCPs’ web-based disclosures on credibility and healthy patient-physician relationship. Their study also emphasizes specifics of SM in a different context, suggesting that the public has expectations of web-based professionalism that warrant further exploration across a range of health professions to broaden our understanding of credibility evaluations in this relation.

The recommendation that HCPs maintain a separate account with a different name, a *dual citizen approach*, which maintains web-based professional and private identities by creating separate web-based profiles was introduced in 2011 [67]. Surprisingly, this issue is still so prevalent [48]. Patients search on the web for their physicians and their impressions about professionalism are based on the publicly available web-based SM content [68,69], but patients also use SM as the most influential web-based method in selecting a physician [69,70]. Besides patients, publicly available content on SM is also screened by future or current employers [48]. This screening is not only performed to make hiring decisions but also to evaluate current employees and assess their behavior and professional competency. Examples exist of unprofessional SM posts as a reason [71] or an alleged reason [72] for firing.

Clear legal violations (HIPAA violations or similar legal transgressions) should and are categorized as unprofessional, but when it comes to potentially unprofessional behavior on SM, it should be judged through the *simulacrum of the professional* only when the context is clearly linked to clinical or work-related settings and to the ability of the individual to practice for the benefit of the patients. This leaves enough room for diversity, self-expression, inclusivity, and equity of the individual.

When the paper by Hardouin et al [22] was retracted with justifications, it has started the #medbikini movement [33,36,37,39,40]. Numerous media articles presented the paper by Hardouin et al [22] as an example of *creepy stalking* [25,73-75], but viewing publicly available information about physicians or HCPs at SM is done by patients or employers, with benefits or consequences, either to their image or their careers. To #medbikini is not potentially unprofessional, yet we should all be aware of publicly available presentations of ourselves we post on SM and how they may affect our professional credibility and integrity, as perception can vary among different groups [65,66].

### Strengths

The sampling method used in this research is one of the main strengths that directly contributes to the quality of obtained data. Systematic sampling creates representative data for a population of interest and reduces the nonresponse bias that other nonprobabilistic sampling (such as a convenient sample) would create. In addition, it has better dispersion control than a random sample.

The SMePROF rubric for assessment of unprofessional content on FB was developed with improved rubric and criteria for unprofessional or potentially unprofessional content, therefore reducing possibilities for subjective interpretation. Through the implementation of these changes, comprehension of e-professionalism was reassessed and updated.

This research is the first assessment of unprofessional behavior on FB that controls for gender bias among coders. In the final results of the second phase (using the SMePROF rubric), there was almost no difference between female and male coders in the coding of potentially unprofessional content or for coding unprofessional content. Thus, we conclude that the SMePROF rubric is a more objective instrument. This is also confirmed by an increase in the gender-based agreement of the final codes for the category professionalism, from 85% (193/227) in the first phase to 96.2% (202/210) in the second phase. Gender-based differences were almost neutralized using the SMePROF rubric.

The reliability of this instrument is mainly demonstrated through reproducibility, with carefully selected independent coders, and further substantiated using a reliability coefficient that increased from Krippendorff α of .61 in the first phase to Krippendorff α of .67 in the second phase. Nonexistence of validated criteria or instruments to assess unprofessional content on FB was emphasized by previous e-professionalism studies [17,19,32]. In content analysis, obeying the translation rules is equivalent to validity [76]. Validity of the coding process is ensured when the researcher is consistent and coherent in their codes, meaning that they follow their translation rules. The SMePROF rubric has face and content validity established through a lengthy development process, containing categories and subcategories identified through a search of the literature and review by interdisciplinary experts followed by further revisions to establish more precise criteria for coding [77].

This research emphasizes the role that context plays in the perception of unprofessional and potentially unprofessional content and provides insight into the existence of a different set of rules for web-based and offline (face-to-face) interactions, which marks behavior as unprofessional.

### Limitations and Future Research

There are several limitations to this study. In the first phase of coding, female coding was conducted before the #medbikini movement, and male coding and mixed-gender coding were conducted after it. This may affect their unconscious and conscious bias during the coding process when analyzing the unprofessional content and potentially unprofessional content. As the #medbikini movement was changing the sensibility of the public and professionals to repressive standards of judging e-professionalism, it could have changed the sensibility of our coders too, resulting in less content coded as potentially unprofessional or unprofessional. The timing of the #medbikini movement that forced us to improve the instrument between the 2 phases also made it difficult to directly demonstrate stability between the 2 repeated measurements.

Secondly, our sample consisted of both phases of the same lists of students and faculty. Students who were sixth year students in the first phase, meantime, finished their education and became MDs or DMDs when the second phase coding was performed. This might affect changes in their privacy settings or affiliation with the school. Our sample has a small total number of faculty representatives from the UZSDM, as it is a much smaller institution than the UZSM, having a total of 166 faculty. Content analysis of faculty’s FB profiles was made by a method of probabilistic systematic sampling of 16.7% of registered faculty, equally distributed according to the academic position and gender, therefore only 28 faculty from the UZSDM entered the final sample for the content analysis versus 86 faculty from the UZSM. Although used systematic sampling offers a nonbiased probabilistic sample, as only 21% (24/114) of the faculty had identifiable FB accounts, conclusions were made based on these results.

For definitive results, if reaching a consensus was not possible between the female or the male coding teams, a third reviewer was consulted, and differences were resolved. The third reviewer was always the same (woman, TVR).

Even though the SMePROF coding scheme demonstrates face and content validity, the construct validity is asserted to be the most valuable indicator of the validity of an instrument established through a practical application over time, demonstrating the instrument’s replicability [77]. As our efforts to enhance the construct validity of the SMePROF coding scheme move forward, we believe that our work on reliability may facilitate the future assessment of the construct validity of this instrument.

The Nason-Koo coding scheme was developed for this study based on previous research [16,20]. Both methodological principles, for the studies by Nason et al [16] and Koo et al [20], were created by MDs or DMDs. The SMePROF coding scheme, especially the criteria for the SMePROF rubric, were developed after the #medbikini movement, with an interdisciplinary team of authors (MDs, DMDs, sociologists, and informational professionals), but we do not have insights from the public or patients about what they consider to be unprofessional or potentially unprofessional behavior. As suggested in previous research [19,65,66], public perceptions about professionalism and credibility are integral to developing the evidence base for e-professionalism assessment, e-professionalism guidelines, and encouraging best practices in SM use. These interventional processes would require multidisciplinary and cross-sectoral input from patients, academic and physician leaders, SM experts, and interprofessional stakeholders [78] that future research should address. The recent systematic review by Guraya et al [79] also calls for assistance and guidance in training the digitally enhanced learning in preparation for their future digitally driven clinical practice. They also emphasize the problem of the multidimensional construct of professionalism, making it hard to assess all domains in the medical field. To add to its complexity, the assessment of e-professionalism is still in its infancy.

We did not include in the coding scheme or assess other SM platforms, such as Instagram, which is highly used among the UZSM and UZSDM students [64]. With the rapid evolution of SM, future insights should be more oriented toward new and emerging SM sites and how different professions among HCPs use them. Kerr et al [80] have explored the characteristics and behaviors of nurses who have attained microcelebrity status on Instagram, but other HCPs show similar tendencies in SM self-promotion, which should be explored more. Instagram has gained enormous popularity by introducing new features such as Stories and Reels, which are completely scientifically unexplored [48]. YouTube as an example of an old SM site, is also unexplored in the context of HCP’s professional behavior, with the study by Lee et al [81], published in 2021, being the first study on digital professionalism behavior on medical students’ YouTube videos. Research shows that students’ perceptions and reports of their Twitter experiences offer insights into behavior on the web and the evolving role of cyberspace, and potentially problematic posts provide opportunities for teaching digital professionalism [82]. Twitter was not included for assessment in our study, as it is not prevalently used by students of medicine or dental medicine in Croatia [64].

The COVID-19 pandemic has caused much of the world’s population to isolate itself and many of us to shift our lives to digital tech platforms, especially SM and SNSs, all experiencing strong growth. Previous research has shown that more people are relying on SM to find and share health information during times of crisis [48]. We are experiencing an unprecedented time in health care and education owing to the COVID-19 pandemic [83], so the use of SM in patient-HCP communication and student education should also be explored in more detail. Examples of sensationalist SM use by MDs and DMDs during the COVID-19 pandemic have been described [84], providing a partial insight as to the likely motivations of physicians and dentists to use SM in a manner that may not necessarily lend well to the professional standards expected. The question of how the pandemic affected our e-professional behavior needs to be explored further.

### Conclusions

Because of this study, the development of a SMePROF coding scheme, a part of which is the SMePROF rubric for the professionalism of HCPs on SM, has reduced the influence of subjective interpretation. Assessment of potentially unprofessional behavior is very subjective. Differences in that assessment may be the result of age, gender, different professional background or level, and other cultural or context related variables. New, more defined evaluation criteria were developed and validated, providing a better instrument for future research. According to the results of this study, the gender of coders did not affect the results for coding unprofessional or potentially unprofessional content using the same methodology and available criteria. This research emphasizes the role that context plays in the perception of unprofessional and potentially unprofessional content and provides insight into the existence of a different set of rules for web-based and offline (face-to-face) interactions that marks behavior as unprofessional.

Finally, the level of web-based professionalism on FB profiles of medical or dental students and faculty available for public viewing has shown a high level of understanding of e-professionalism, with unprofessional content being very low. This is indicative of the new more open view of professionalism on SM that will continue to evolve in the years to come.

## Appendix

Multimedia Appendix 1 Twitter Binder report: #medbikini historical search for the period from September 9, 2013 to January 16, 2022.

Multimedia Appendix 2 Health care professionals’ social media engagement related to the #medbikini movement.

Multimedia Appendix 3 Instruments used in the first phase of the study.

Multimedia Appendix 4 Main differences comparing the Koo rubric and SMePROF rubric for assessment of unprofessional content on Facebook.

Multimedia Appendix 5 Overview of previous research descriptions of potentially questionable, unprofessional, or objectionable behavior on social media.

## Abbreviations

DMD: doctor of dental medicine
FB: Facebook
HCP: health care professional
HIPAA: Health Insurance Portability and Accountability Act
ICR: intercoder reliability
MD: doctor of medicine
ReCal: Reliability Calculator
SM: social media
SNS: social networking site
UZSDM: School of Dental Medicine University of Zagreb
UZSM: School of Medicine University of Zagreb
