# Supplementary material for: Defining Potentially Unprofessional Behavior on Social Media for Health Care Professionals: Mixed Methods Study
Source: JMIR Med Educ. 2022 Aug 9;8(3):e35585. doi: 10.2196/35585 (PMC9399843; doi:10.2196/35585)
Supplement: Multimedia Appendix 1 [file mededu_v8i3e35585_app1.pdf]

#medbikini

TIME PERIOD 9/18/2013 - 1/16/2022

SUMMARY

TWEETS: 60,002 • USERS: 40,863

60,002
Total tweets

459,782.14 $ **economic value**

50.36 % **sentiment score**

TEXT TWEETS	**4.67%**	2,805
REPLIES	**7.24%**	4,343
RETWEETS	**76.19%**	45,716
LINKS AND IMAGES	**13.82%**	8,292

227,048,455 **potential impacts**	114,583,415 **potential reach**	40,863 **total contributors**	1.47 **tweets per contributor**
2,804.09 **followers per contributor**	14,286 **original tweets**	10,349 **original contributors**	1.38 **original tweets per contributor**

This is a Multimedia Appendix to a full manuscript published in the JMIR Med Educ. For full copyright and citation information see http://dx.doi.org/10.2196/35585

#medbikini

TIME PERIOD 9/18/2013 - 1/16/2022

TIMELINE

• TWEETS: 60,002 • USERS: 40,863

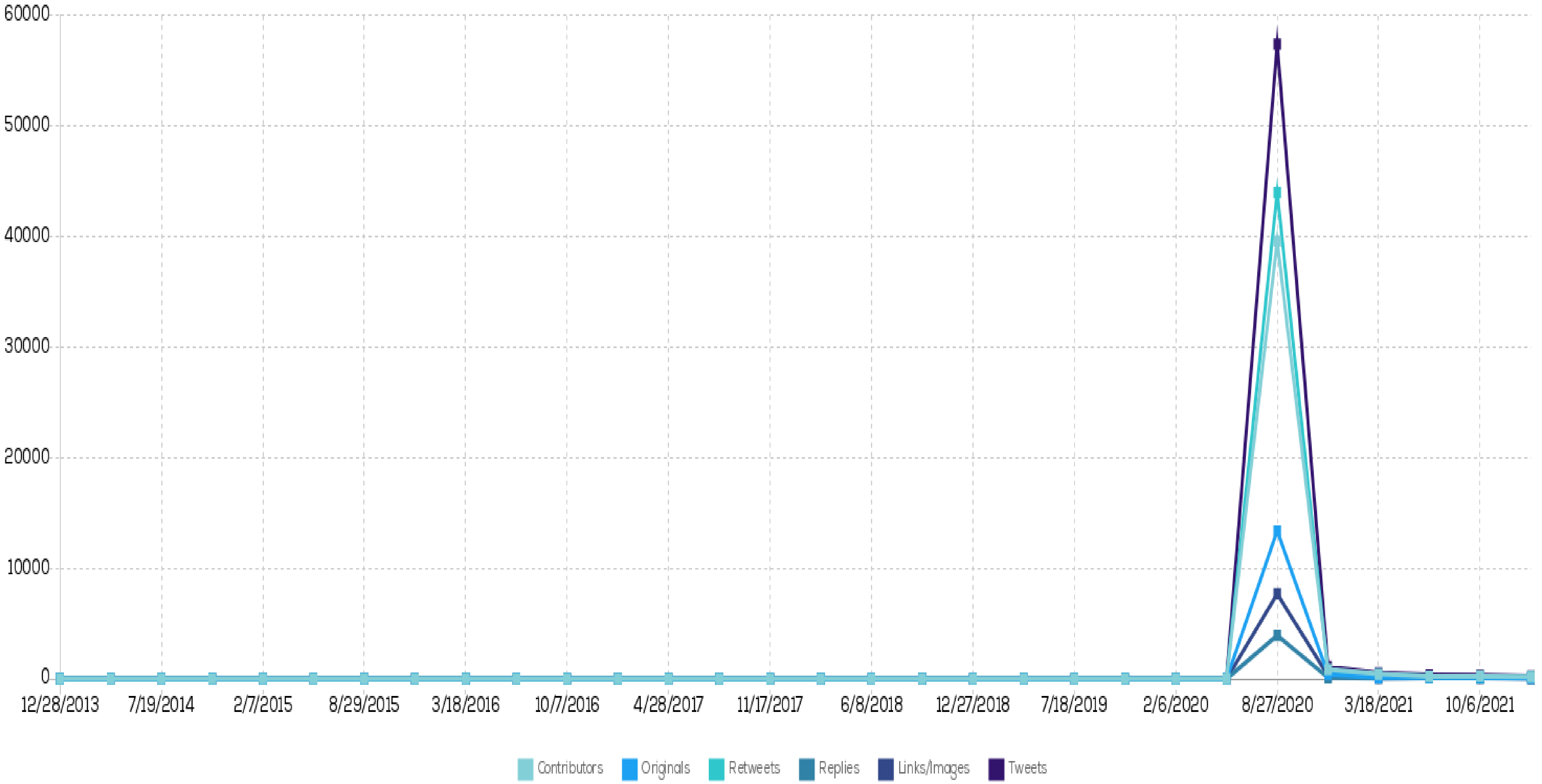

#medbikini

TIME PERIOD 9/18/2013 - 1/16/2022

MOST RETWEETED TWEETS

• TWEETS: 60,002 • USERS: 40,863

Madeeha_Syed | 1 year ago | 10642 Retweets
This is the most badass #MedBikini post I've read and I'm sorry if Dr. Candice Myhre is on twitter and shared this. But I think we all need to see and read it. Wearing a bikini did not take away from her duties as a doctor. It didn't hurt her pt. It actually saved their life. https://t.co/rze0afLksT

DrChowdharyMD | 1 year ago | 3317 Retweets
If you are a true #heforshe then you must speak up against this disturbing study 3 men created fake social media accounts to purposefully spy on applicants Worse they are shaming our women physician colleagues for wearing bikinis ??? #MedTwitter #MedBikini #retraction https://t.co/MvNZoBnok2

NickLeighton12 | 1 year ago | 1297 Retweets
So this study was published shaming physicians for being "unprofessional" by wearing bikinis or holding a beer in a photo? And the study was conducted by 3 men who created fake social media accounts to spy on applicants? This "study" must be retracted. #MedTwitter #MedBikini https://t.co/h3SXs3YXUO

DGlaucomflecken | 1 year ago | 1144 Retweets
The #medbikini movement is one of the best medtwitter things I've ever seen. Doctors are people who do normal people things. Big thanks to the horribly misguided and borderline unethical vascular surgery publication that made it all possible.

ProfPCDoherty | 1 year ago | 1028 Retweets
#Insiders There's too much on death counts in COVID-19. This is a new virus with a substantial vascular & inflammatory component. We have absolutely no understanding of possible long-term health consequences. We do know that many younger people are suffering long-term debility.

DrDooleyMD | 1 year ago | 1011 Retweets
Apparently it's unprofessional for doctors to post social media pics wearing bikinis & drinking alcohol, so here's me doing both. #MedBikini Also deemed unprofessional, profanity & political talk but these dudes can fuck off b/c health care should be free at the point of care. https://t.co/Lk99jCRdrD

stephlococcus | 1 year ago | 689 Retweets
I am a woman in medicine who loves to travel to tropical locations and dress accordingly. I will not wear my white coat and scrubs to Hawaii. This does not make me unprofessional or less intelligent or compassionate compared to my male colleagues. #medbikini #girlmedtwitter https://t.co/RmCQBnUme6

#medbikini

TIME PERIOD 9/18/2013 - 1/16/2022

MOST LIKED TWEETS

TWEETS: 60,002 • USERS: 40,863

Madeeha_Syed | 1 year ago | 35129 Likes

This is the most badass #MedBikini post I've read and I'm sorry if Dr. Candice Myhre is on twitter and shared this. But I think we all need to see and read it. Wearing a bikini did not take away from her duties as a doctor. It didn't hurt her pt. It actually saved their life. https://t.co/rze0afLksT

DrDooleyMD | 1 year ago | 10299 Likes

Apparently it's unprofessional for doctors to post social media pics wearing bikinis & drinking alcohol, so here's me doing both. #MedBikini Also deemed unprofessional, profanity & political talk but these dudes can fuck off b/c health care should be free at the point of care. https://t.co/Lk99jCRdrD

trishgreenhalgh | 1 year ago | 9179 Likes

Ha! Found a selfie in a bikini. To the 28 year old "researcher" who says this is unprofessional for women doctors, I'm old enough to be your grandmother. #MedBikini https://t.co/84CKW9nfhz

DrChowdharyMD | 1 year ago | 8168 Likes

If you are a true #heforshe then you must speak up against this disturbing study 3 men created fake social media accounts to purposefully spy on applicants Worse they are shaming our women physician colleagues for wearing bikinis ��� #MedTwitter #MedBikini #retraction https://t.co/MvNZoBnok2

meganedurham | 1 year ago | 13096 Likes

I've thought long and hard about posting my #MedBikini contribution, because so many of them don't look like me. But I'm a firm believer that the only requirement for a bikini body is to put on a bikini. #everybodyisabikinibody https://t.co/zqanuxksNj

stephlococcus | 1 year ago | 10082 Likes

I am a woman in medicine who loves to travel to tropical locations and dress accordingly. I will not wear my white coat and scrubs to Hawaii. This does not make me unprofessional or less intelligent or compassionate compared to my male colleagues. #medbikini #girlmedtwitter https://t.co/RmCQBnUme6

DGlaucomflecken | 1 year ago | 8791 Likes

The #medbikini movement is one of the best medtwitter things I've ever seen. Doctors are people who do normal people things. Big thanks to the horribly misguided and borderline unethical vascular surgery publication that made it all possible.

#medbikini

SENTIMENT ANALYSIS

TIME PERIOD 9/18/2013 - 1/16/2022

• TWEETS: 60,002 • USERS: 40,863

50.36

sentiment score

by number of users

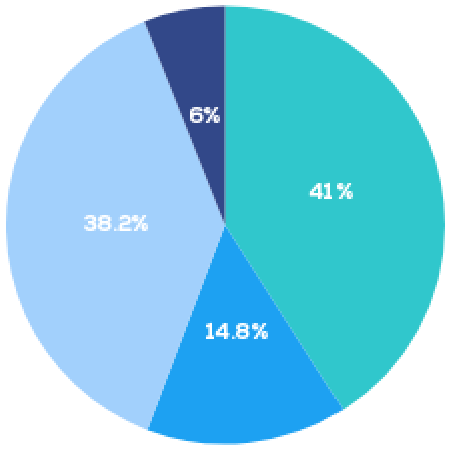

by number of tweets

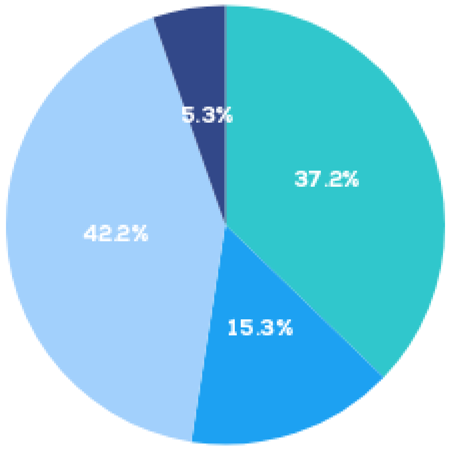

by number of impacts

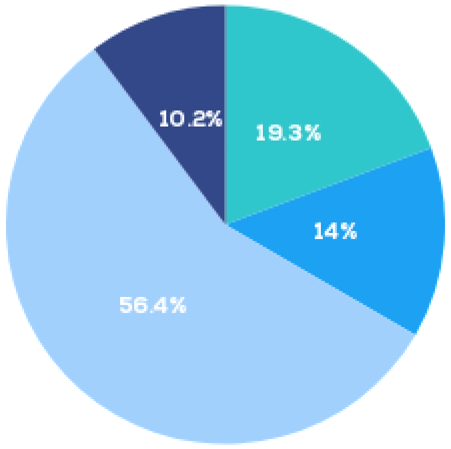

POSITIVE NEUTRAL NEGATIVE UNDEFINED

#medbikini

TIME PERIOD 9/18/2013 - 1/16/2022

SENTIMENT ANALYSIS TIMELINE

TWEETS: 60,002 • USERS: 40,863

sentiment analysis timeline

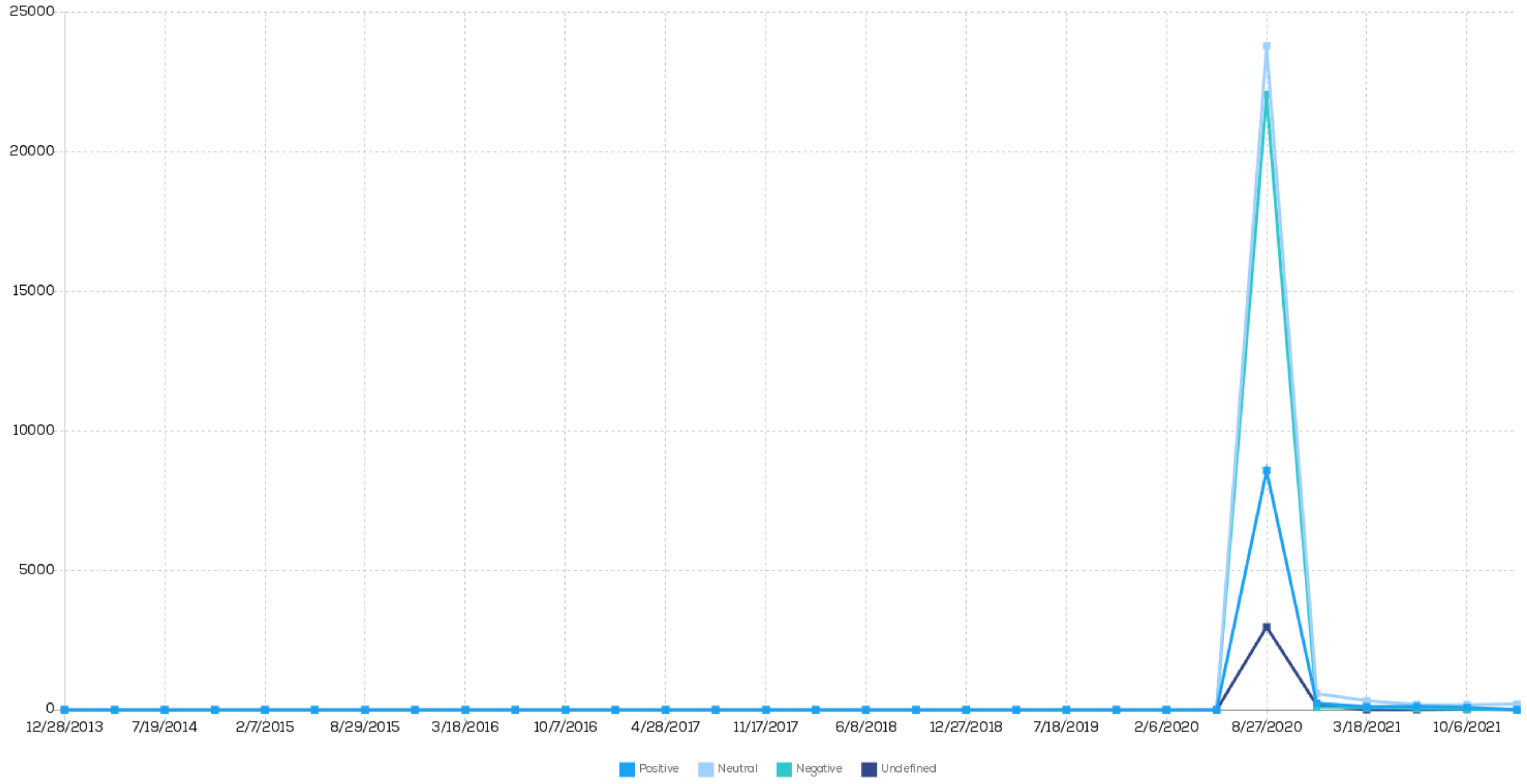

sentiment statistics

	TOTAL TWEETS	POTENTIAL IMPACTS	CONTRIBUTORS	ECONOMIC VALUE	LINKS/ PICS	RETWEETS
NEUTRAL	25,316	128,120,255	18,126	236,502.8	4,591	17,563
POSITIVE	9,168	31,897,661	7,012	48,142.07	1,551	6,553
NEGATIVE	22,318	43,848,863	19,422	70,905.84	1,207	20,101

#medbikini

TIME PERIOD 9/18/2013 - 1/16/2022

CONTRIBUTOR RANKINGS

TWEETS: 60,002 • USERS: 40,863

MOST ACTIVE	Tweets
@usnehal Umbereen S...	264
@IBTimesHindi IBTimes Pub...	175
@londyloo Londyn Robi...	147
@BRob2018 Brandon Ro...	111
@olearypd Pamela O'Le...	99

RETWEETERS	Retweets
@BRob2018 Brandon Ro...	110
@olearypd Pamela O'Le...	91
@pattybarrue Patty Barrué	76
@BrittGratreak Britt DK Grat...	75
@Ad31__ ��AD31 Éter...	60

ORIGINAL TWEETS	Tweets
@usnehal Umbereen S...	236
@IBTimesHindi IBTimes Pub...	171
@londyloo Londyn Robi...	92
@IamSaifRK Saif �	42
@geno_md Geno Tai MD...	38

MOST POPULAR	Followers
@Independent The Indepen...	3,303,472
@CBCNews CBC News	3,205,127
@RehamKhan1 Reham Khan	2,645,741
@bbcbrasil BBC News B...	2,626,539
@canalgnt Canal GNT	2,453,265

HIGHEST IMPACT	Impacts
@Independent The Indepen...	23,124,301
@canalgnt Canal GNT	4,906,530
@CBCNews CBC News	3,205,127
@ABC7Chicago ABC 7 Chica...	3,159,484
@threadreaderapp Thread Rea...	2,647,609

TOP PHOTOGRAPHER	Photos
@usnehal Umbereen S...	70
@TomthunkitsMind Tomthunkit™	35
@IndiesUnite Filmmakers | ...	32
@JerryBeller1 The Right W...	21
@WokeDoctors WokeDoctor...	13

#medbikini

CONTRIBUTOR RANKINGS

TIME PERIOD 9/18/2013 - 1/16/2022

TWEETS: 60,002 • USERS: 40,863

VERIFIED USERS	Followers
@Independent The Indepen...	3,303,472
@CBCNews CBC News	3,205,127
@RehamKhan1 Reham Khan	2,645,741
@bbcbrasil BBC News B...	2,626,539
@canalgnt Canal GNT	2,453,265

TOP RTS ENGAGERS	RTs
@Madeeha_Syed Madeeha_S...	5,323
@DGlaucomflecken Dr. Glaucomf...	650
@mirlenny2426 Miriam Guerr...	646
@08escaper หัวฟูกé	561
@LGlaucomflecken Lady Glauco...	481

TOP LIKES ENGAGERS	Likes
@Madeeha_Syed Madeeha_S...	17,630.5
@DGlaucomflecken Dr. Glaucomf...	7,182.5
@meganedurham MED, M.D.	6,848
@LGlaucomflecken Lady Glauco...	5,461
@KesiaNguyen Kesia	3,736

MOST RETWEETED	Retweets
@Madeeha_Syed Madeeha_S...	10,646
@DrChowdharyMD Mudit Chow...	3,557
@ProfPCDoherty Prof. Peter ...	1,521
@NickLeighton12 Nicolas Leig...	1,328
@DGlaucomflecken Dr. Glaucomf...	1,300

MOST FAVORITED	Likes
@Madeeha_Syed Madeeha_S...	35,261
@DGlaucomflecken Dr. Glaucomf...	14,365
@meganedurham MED, M.D.	13,696
@trishgreenhalgh Trisha Green...	10,974
@DrDooleyMD Dr. Victoria D...	10,675

MOST MENTIONED	Mentions
@jvascsurg J Vascular S...	398
@londyloo Londyn Robi...	85
@choo_ek Esther Choo ...	72
@	63
@drchowdharymd Mudit Chow...	59

#medbikini

459,782.14 $

report's economic value

The economic value of the hashtag or term analyzed is calculated after analyzing each one of the contributors (followers, following, list, etc.) and their tweets. This shows how much the hashtag is worth.

7.52 $
average user's value

7.66 $
average tweet's value

economic tweet's value

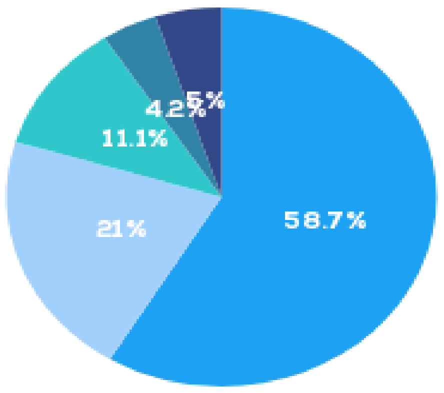

economic user's value

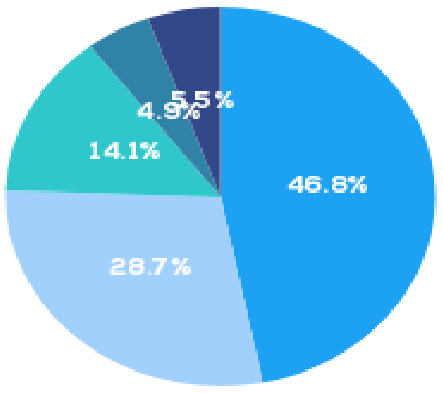

- **XS:** 0.00 $ - 1.14 $
- **S:** 1.14 $ - 3.42 $
- **M:** 3.42 $ - 9.13 $
- **L:** 9.13 $ - 17.12 $
- **XL:** >= 17.12 $

#medbikini

TIME PERIOD 9/18/2013 - 1/16/2022

USERS RANKING

TWEETS: 60,002 • USERS: 40,863

TOP ACCOUNTS	Value
@Independent The Indepen...	6,953.29 $
@CBCNews CBC News	6,557.85 $
@bbcbrasil BBC News B...	5,548.34 $
@RehamKhan1 Reham Khan	5,515.92 $
@FastCompany Fast Compa...	5,365.55 $
@canalgnt Canal GNT	5,143.17 $
@ClevelandClinic Cleveland Cli...	4,303.20 $
@glamourmag Glamour	3,030.19 $
@digg Digg	2,765.82 $
@ABC7 ABC7 Eyewi...	2,621.60 $

MOST EXPENSIVE USERS	Value
@Independent The Indepen...	53,540.36 $
@canalgnt Canal GNT	12,343.62 $
@ABC7Chicago ABC 7 Chica...	9,477.18 $
@TomthunkitsMind Tomthunkit™	7,749.80 $
@abc13houston ABC13 Hous...	7,355.48 $
@CBCNews CBC News	7,213.63 $
@kevinmd Kevin Pho, M...	7,032.14 $
@ABC7NY Eyewitness ...	6,608.22 $
@bbcbrasil BBC News B...	6,103.18 $
@RehamKhan1 Reham Khan	6,067.51 $

#medbikini

TIME PERIOD 9/18/2013 - 1/16/2022

TWEETS' ECONOMIC VALUE

• TWEETS: 60,002 • USERS: 40,863

Independent | 1 year ago | 6699.85 €
Hundreds of doctors are responding to sexist study by flooding the internet with bikini pictures https://t.co/rqnRzozNr5

Independent | 1 year ago | 6699.85 €
Hundreds of doctors are responding to sexist study by flooding the internet with bikini pictures https://t.co/hINIL2Ox3g

Independent | 1 year ago | 6699.85 €
Hundreds of doctors are responding to sexist study by flooding the internet with bikini pictures https://t.co/hINIL2Ox3g

Independent | 1 year ago | 6699.85 €
Hundreds of doctors are responding to sexist study by flooding the internet with bikini pictures https://t.co/rqnRzoRoiD

Independent | 1 year ago | 6699.85 €
Hundreds of doctors are responding to sexist study by flooding the internet with bikini pictures https://t.co/gWKdwQN7Jk

Independent | 1 year ago | 6699.85 €
Now surgeons are being judged at work by what they wear on their holidays. That's sexism | Harriet Hall https://t.co/cSaSQmhhvP

Independent | 1 year ago | 6699.85 €
Hundreds of doctors are responding to sexist study by flooding the internet with bikini pictures https://t.co/hINIL2Ox3g

#medbikini

TIME PERIOD 9/18/2013 - 1/16/2022

INFLUENCE STATS

TWEETS: 60,002 • USERS: 40,863

TWEETS/ CONTRIBUTORS

1.47 tweets/ contributor

TWEETS	CONTRIBUTORS	% TOTAL
1	33439	81.83%
2	4245	10.39%
3	1427	3.49%
4	616	1.51%
5	369	0.9%
6	177	0.43%
>=7	590	1.44%

CONTRIBUTOR INFLUENCE

2,804.09 followers/ contributor

FOLLOWERS	CONTRIBUTORS	% TOTAL
XXS (0-10)	1162	2.84%
XS (10-50)	3464	8.48%
S (50-200)	9180	22.47%
M (200-500)	10088	24.69%
L (500-1000)	6651	16.28%
XL (1000-5000)	7801	19.09%
XXL (5000-∞)	2517	6.16%

AGE OF THE TWITTER ACCOUNTS

YEARS	USERS	% TOTAL
XXS (0-1)	44	0.11%
XS (1-2)	3276	8.02%
S (2-3)	3163	7.74%
M (3-4)	2599	6.36%
L (4-5)	2413	5.91%
XL (5-6)	2374	5.81%
XXL (6-∞)	26994	66.06%

LENGTH OF THE TWEETS

171.09 characters/ tweet

CHARACTERS	TWEETS	% TOTAL
XXS (0-40)	919	6.43%
XS (40-80)	1863	13.04%
S (80-120)	2119	14.83%
M (120-160)	1798	12.59%
L (160-200)	1609	11.26%
XL (200-240)	1697	11.88%
XXL (240-∞)	4281	29.97%

#medbikini

TIME PERIOD 9/18/2013 - 1/16/2022

ADDITIONAL RANKINGS

• TWEETS: 60,002 • USERS: 40,863

TOP LANGUAGES

LANGUAGE	TWEETS
ENGLISH	53231
SPANISH	3339
PORTUGUESE	1216
UNDETERMINED	949
JAPANESE	240
FRENCH	210
THAI	207
RUSSIAN	112
DUTCH	105
GERMAN	62

TOP SOURCES

SOURCE	TWEETS
TWITTER FOR IPHONE	31463
TWITTER FOR ANDROID	14257
TWITTER WEB APP	10889
TWITTER FOR IPAD	1526
TWEETDECK	270
HOOTSUITE INC.	136
INSTAGRAM	135
TWEETBOT FOR IOS	122
DLVR.IT	121
BUFFER	78

TOP HASHTAGS

HASHTAG	TWEETS
#MEDBIKINI	50059
#MEDTWITTER	9445
#HEFORSHE	3632
#RETRACTION	2949
#PROFESSIONALISM	1414
#INSIDERS	976
#MEDKINI	559
#MEDED	550
#WOMENINMEDICINE	506
#GIRLMEDTWITTER	490
