# Supplementary material for: Defining Potentially Unprofessional Behavior on Social Media for Health Care Professionals: Mixed Methods Study
Source: JMIR Med Educ. 2022 Aug 9;8(3):e35585. doi: 10.2196/35585 (PMC9399843; doi:10.2196/35585)
Supplement: Multimedia Appendix 2 [file mededu_v8i3e35585_app2.pdf]

Healthcare professionals' social media engagement related to the #medbikini movement

On January 15th 2022, there are 10.000 posts on Facebook [26]. and 27.911 on Instagram [27] related to #medbikini.

Image 1. Dr. Londyn Robinson's tweet that started the #medbikini movement, July 23rd 2020 [23].

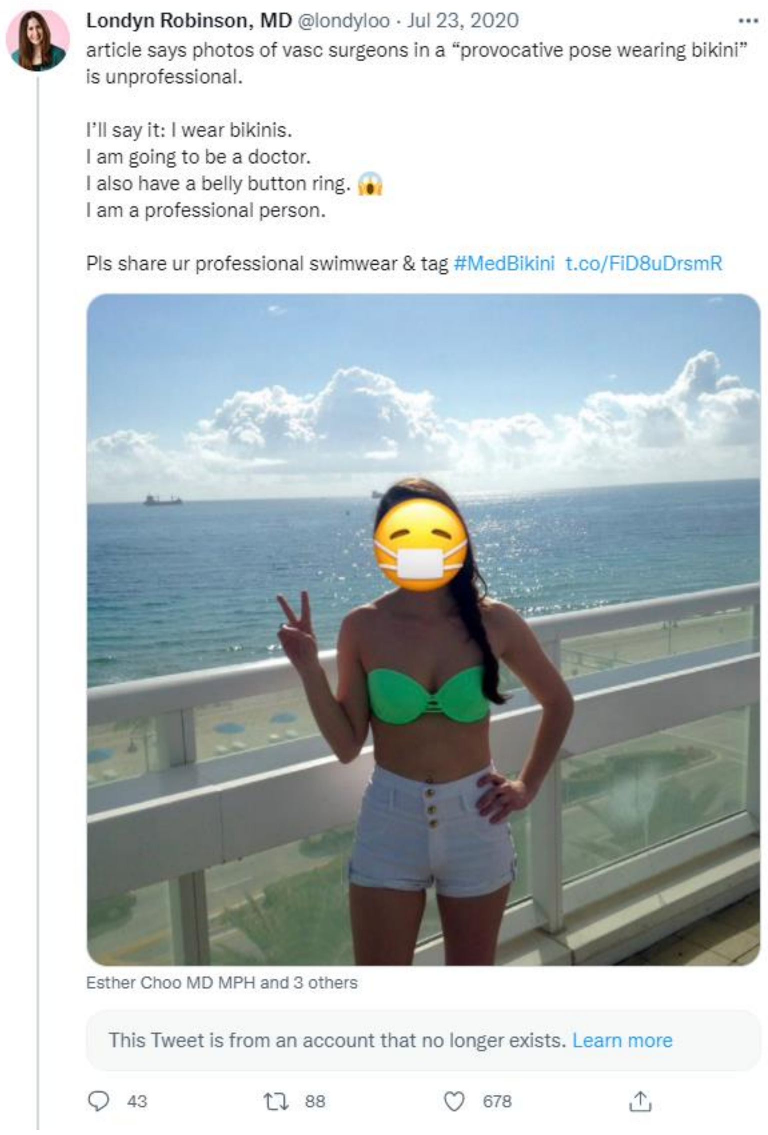

Examples of HCPs social media reactions related to the #medbikini movement:

Image 2. No one dictates what I can and cannot wear [28].

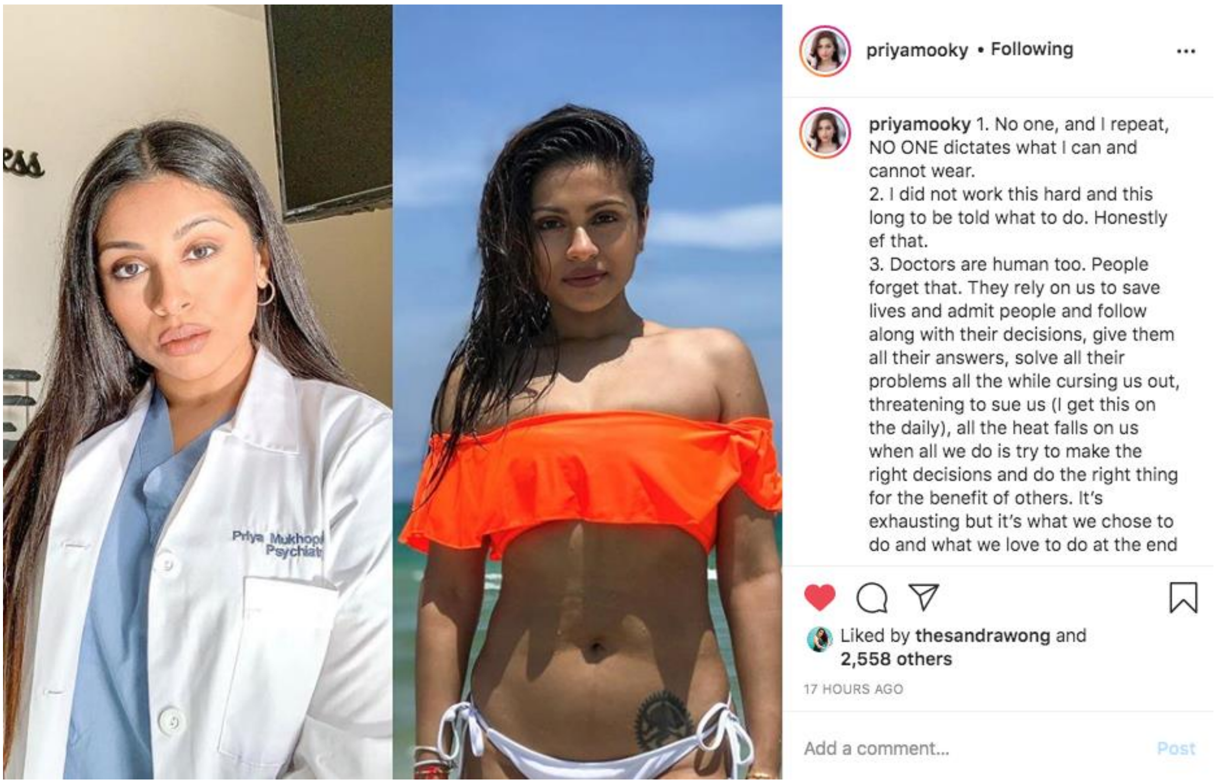

Image 3. Surgeons can wear bikinis [28].

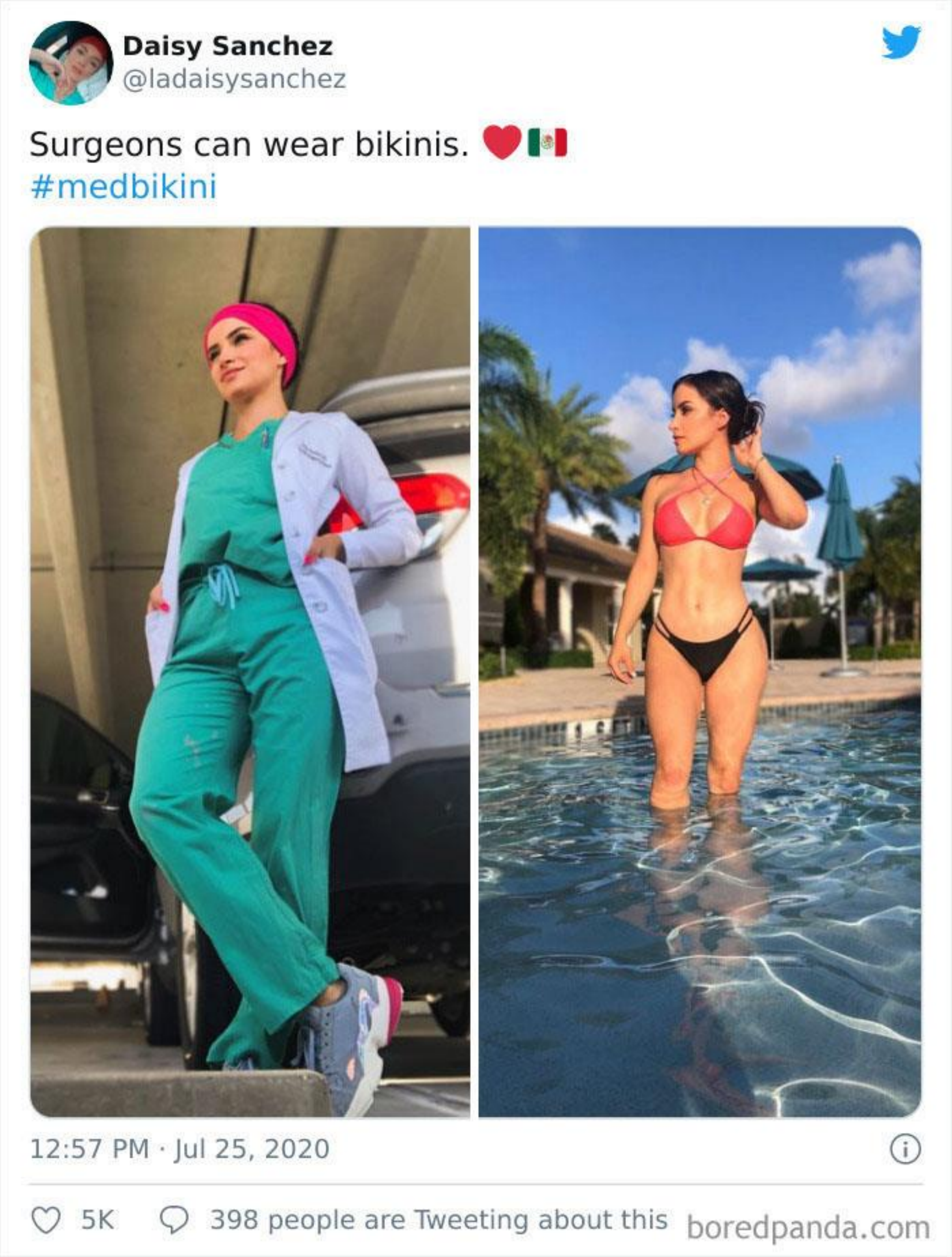

Image 4. A woman can have beauty and brains [28].

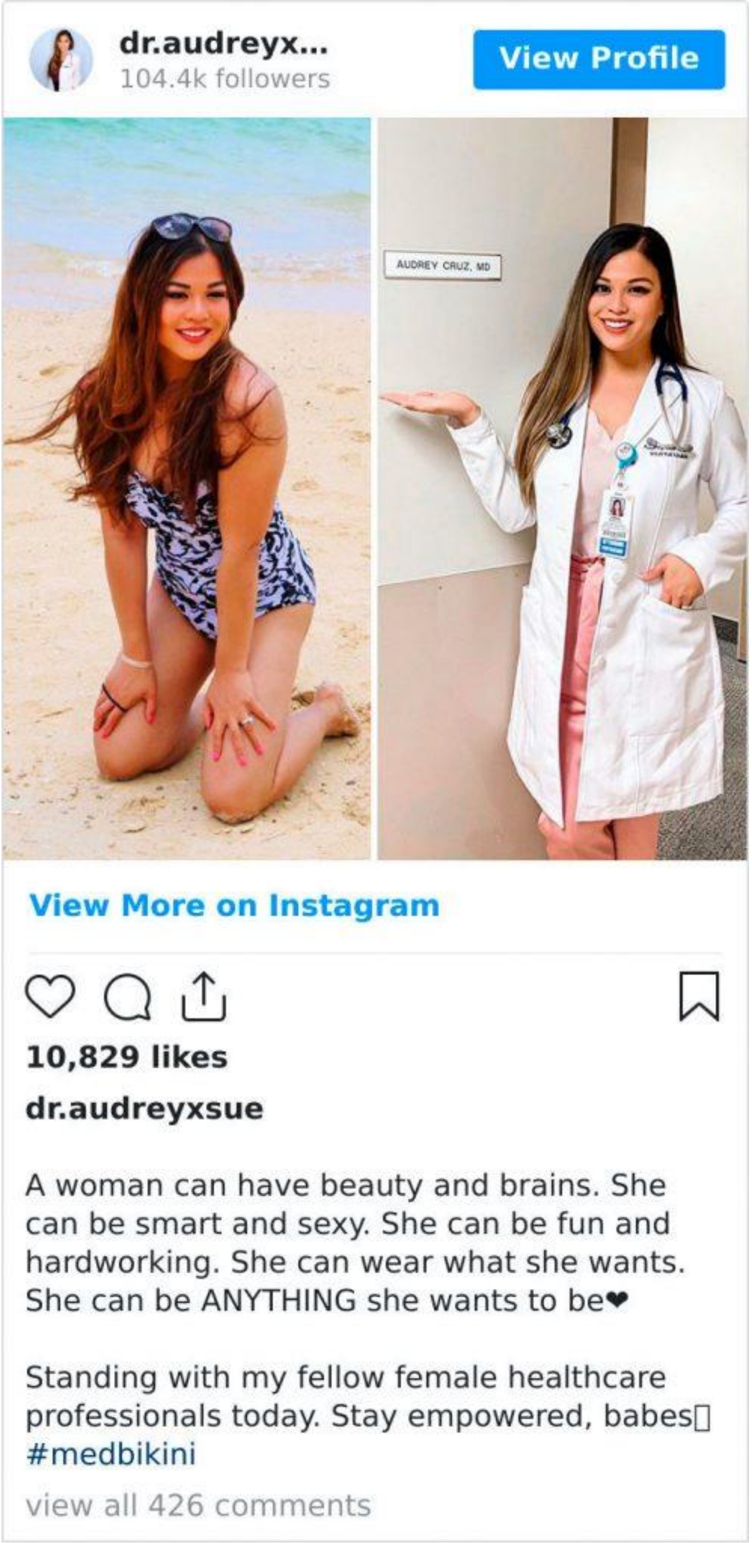

Image 5. Call for a retraction of the paper [29].

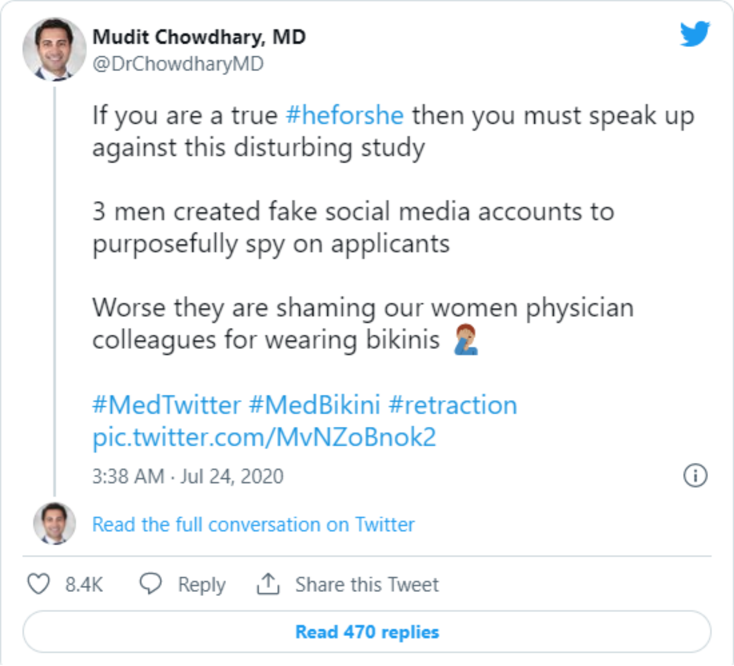

Image 6. Couple of “unprofessional” obstetrician gynecologists [30].

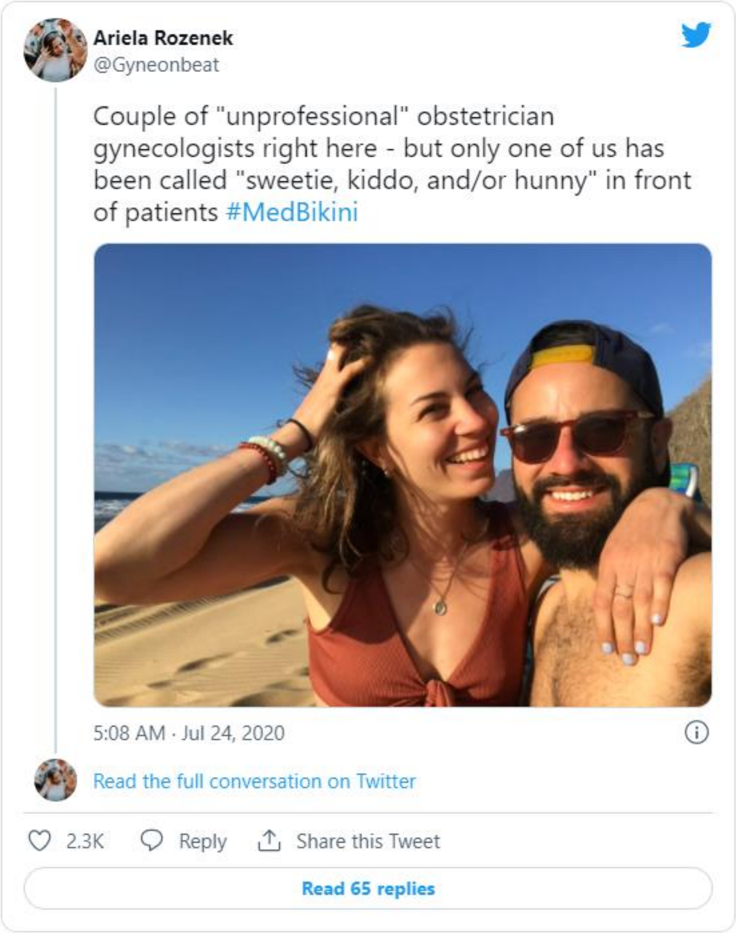

This is a Multimedia Appendix to a full manuscript published in the JMIR Med Educ. For full copyright and citation information see http://dx.doi.org/10.2196/35585.
