# Supplementary material for: Defining Potentially Unprofessional Behavior on Social Media for Health Care Professionals: Mixed Methods Study
Source: JMIR Med Educ. 2022 Aug 9;8(3):e35585. doi: 10.2196/35585 (PMC9399843; doi:10.2196/35585)
Supplement: Multimedia Appendix 3 [file mededu_v8i3e35585_app3.pdf]

Instruments used in the first phase of the study: Nason-Koo coding scheme

Nason-Koo coding scheme [16, 20-21] consists of six categories previously used in Nason et al study [16]: 1) existence of identifiable FB profile, 2) sex, 3) privacy settings, 4) relationship status revealed, 5) affiliation with the school revealed and 6) professionalism.

For determining existence of *identifiable FB profile*, name search from neutral FB account was performed. Name searches that returned multiple results were narrowed down by network, affiliation, institution or current location to identify the correct profile. If a name search returned multiple results and could not be narrowed down, it was excluded from the study and classified as "impossible to determine".

If identifiable FB profile was found, it was included in the study and *sex* was determined by name and picture. In category *privacy settings*, identifiable Facebook profile was categorized as private or public. A private profile was defined as one that contained only basic demographic information that did not extend beyond age, educational history and location. If additional information (interests, pages, photos, posts, quotes, relationship status, etc.) was provided, it was considered to be a public profile.

Details including educational *relationship status*, mention of being a medical/dental student or being a faculty member, mention of, or any kind of *affiliation with the medical/dental school* were recorded for each individual Facebook profile.
Each profile was subsequently scored with regard to *professionalism*, based on Koo et al's rubric for assessment of unprofessional content on Facebook (Koo rubric) [20,21] (Table 1.)

Table 1. Nason-Koo coding scheme.

Category	Codes
**Facebook profile**	
	identified
	impossible to determine
	does not have a FB profile
**Sex**	
	male
	female
**Privacy settings**	
	public
	private
**Relationship status revealed**	
	yes
	no
**Affiliation with the School revealed**	
	yes
	no
**Professionalism**[a]	
	unprofessional content
	potentially objectionable content
	professional content

[a]assessed using Koo et al's rubric for assessment of unprofessional content on Facebook

Category professionalism was coded according to the Koo et al's rubric for assessment of unprofessional content on Facebook (Koo-rubric) [20,21] (Table 2). Each account was reviewed for 35 possible sub-categories of unprofessional or potentially objectionable content using a prospectively designed rubric based on online professionalism guidelines). Profiles were categorized as: a) unprofessional content if at least one element of unprofessional content was found, b) potentially objectionable content if at least one element of potentially objectionable content was found or c) professional content if none of the elements of unprofessional nor potentially objectionable content were found. Compared to the original Koo et al rubric [20,21], the only change was made for the potentially objectionable content sub-category inappropriate or offensive attire. The sub-category offensive attire (for an example image of a person with uncensored profanity text on a T-shirt) was categorized as unprofessional content. The sub-category inappropriate attire was coded as potentially objectionable content.

Table 2. Koo et al's rubric for assessment of unprofessional content on Facebook (Koo rubric).

Content Type	Unprofessional Content	Potentially Objectionable Content
Image		
	Protected health information	Holding alcohol
	Unprofessional behavior at work or in a professional capacity (eg, conference)	Consuming alcohol
	Offensive attire[a]	Inappropriate attire[a]
	Engaging in unlawful behavior	Appearing in sexually suggestive attire or circumstances
	Possession of drugs or appearance thereof	Politics or content of a political nature
	Displaying drug paraphernalia	Religion or content of a religious nature
	Appearing intoxicated	Controversial or polarizing social topics (e.g., gun control, abortion)
Text		
	Protected health information	Censored profanity
	References to specific instances of unprofessional behavior at work or in a professional capacity	Politics or comments of a political nature
	References to specific instances of unlawful behavior	Religion or comments of a religious nature
	References to possession of drugs	Comments about controversial or polarizing social topics
	References to drug paraphernalia	References to specific instances of sex or sexual behavior
	References to specific instances of alcohol intoxication	
	Uncensored profanity	
	Offensive comments about colleagues at own hospital	
	Offensive comments about colleagues at other hospitals	
	Offensive comments about a specific patient	
Page, link, or other posted content		
	Advocating or supporting the use of drugs	Politics or content of a political nature
	Advocating or supporting unlawful behavior	Religion or content of a religious nature

		Controversial or polarizing social topics
		Advocating or supporting alcohol intoxication
		Sex or sexual behavior

[a]difference compared to original Koo's rubric

This is a Multimedia Appendix to a full manuscript published in the JMIR Med Educ. For full copyright and citation information see http://dx.doi.org/10.2196/35585.
