# Supplementary material for: Defining Potentially Unprofessional Behavior on Social Media for Health Care Professionals: Mixed Methods Study
Source: JMIR Med Educ. 2022 Aug 9;8(3):e35585. doi: 10.2196/35585 (PMC9399843; doi:10.2196/35585)
Supplement: Multimedia Appendix 4 [file mededu_v8i3e35585_app4.pdf]

Main differences comparing the Koo rubric and SMEPROF rubric for assessment of unprofessional content on Facebook

The SMePROF rubric includes more precise definitions of sub-categories, and exclusion of the sub-categories that authors do not consider to be the basis for unprofessional content or potentially unprofessional content (Table 1).

Table 1. Main differences comparing the Koo rubric and SMEPROF rubric for assessment of unprofessional content on FB.

Excluded sub-categories of unprofessional/potentially objectionable content (from Koo rubric)		New sub-categories or re-defined subcategories of unprofessional/potentially unprofessional content with more precise criteria (in SMEPROF rubric)	
**Unprofessional content**	**Potentially objectionable content**	**Unprofessional content**	**Potentially unprofessional content**
Unprofessional behavior at work or in a professional capacity (e.g. conference)			
References to specific instances of unprofessional behavior at work or in a professional capacity			
	Holding alcohol	Holding or consuming alcohol in a clinically/work related setting	
	Consuming alcohol	Holding or consuming alcohol in a clinically/work related setting	
	Inappropriate or offensive attire	Offensive attire	Inappropriate attire
	Appearing in sexually suggestive attire or circumstances		Sexualization
	References to specific instances of sex or sexual behavior		Reference to sexually provocative or sexually disturbing content (either as image, text, page, link or other posted content)
	Politics or content/comments of a political nature	Offensive content of a political, religious, or racial nature (either as image, text, page, link	

		or other posted content)	
	Religion or content/comments of a religious nature	Offensive content of a political, religious, or racial nature (either as image, text, page, link or other posted content)	
	Comments about controversial or polarizing social topics (e.g., gun control, abortion; either as image, text, page, link or other posted content)	Offensive content of a political, religious, or racial nature (either as image, text, page, link or other posted content)	

We considered sub-categories, images of unprofessional behavior at work or in a professional capacity (e.g., conference), or references to specific instances of unprofessional behavior at work or in a professional capacity were too general, and already covered by all other sub-categories, so they were excluded. We have excluded sub-categories for potentially unprofessional content consuming/holding alcohol, if it is not related to a clinical/work related setting. We consider controversial or polarizing social topics (e.g., gun control, abortion) or comments about controversial or polarizing social topics (either as image, text, page, link or other posted content) as a right of free speech, as long they are not offensive. If they are offensive, then they should be categorized as unprofessional content.

The SMePROF rubric differentiates offensive attire vs. inappropriate attire: Offensive attire is defined as photo or video content of an attire that includes offensive elements, for example, wearing a T-shirt with profanity or Nazi symbols (work or non-work related). Inappropriate attire is defined as photo or video content in a clinical/work environment in which an individual is wearing physicians' attire (lab coat, scrubs, surgical gowns, etc.) and also partially revealing skin (sleeveless, deep cleavage, abdomen, back, short pants, or skirts high above the knee) or underwear inappropriate for clinical/work environment. Even though SM allows us to present ourselves in a more private and intimate way (if we allow it using the privacy settings), publicly available images or videos related to clinical/work settings still require higher standards of appearance, including physicians' clothing/attire.

We propose a new sub-category *sexualization* in the potentially unprofessional behavior, with an explanation that sexualization focuses on sexual suggestive/provocative posing (in a professional or private setting), regardless of the attire or revealing clothing, excluding non-sexual suggestive posing in swim/beachwear. Previous research [17,18,20–22], have not clearly defined it, which led to broad possibilities for interpretation of this category, also resulting in the #medbikini movement. [22,33,36,37,39,40].

This is a Multimedia Appendix to a full manuscript published in the JMIR Med Educ. For full copyright and citation information see http://dx.doi.org/10.2196/35585.
