# Supplementary material for: Defining Potentially Unprofessional Behavior on Social Media for Health Care Professionals: Mixed Methods Study
Source: JMIR Med Educ. 2022 Aug 9;8(3):e35585. doi: 10.2196/35585 (PMC9399843; doi:10.2196/35585)
Supplement: Multimedia Appendix 5 [file mededu_v8i3e35585_app5.pdf]

Overview of previous research descriptions of potentially questionable, unprofessional, or objectionable behavior on social media

The first paper that tried to describe questionable content on SNSs was from 2013 by Ponce et al's [14], where questionable content was described as only one example: "may have been evidence of public intoxication", based on AMA and Accreditation Council for Graduate Medical Education (ACGME) regulation from 2009 [6,50].

Langenfeld et al [17], in 2014, as a part of the potentially unprofessional category introduced the term questionable attire (including costumes and revealing swimwear), referring to ACGME description [51] and AMA's report on professionalism in the use of SM [6], as frameworks for the creation of this category. Their description of clearly unprofessional content was described as follows: HIPAA violation, inappropriate language, picture or reference to binge drinking, drug use, racist or sexist content, and sexually suggestive material. Examples of sexually suggestive photos included simulated oral sex, female residents in bikinis with their hands pointing to their breasts, and a female resident simulating intercourse with a large cannon. The description of potentially unprofessional content included: alcohol or tobacco in hand, questionable attire (including costumes and revealing swimwear), polarizing political or religious statements, and weapons [17]. In this paper, it is unclear whether a bikini also refers to revealing swimwear, as the term bikini was specifically mentioned only as part of the clearly unprofessional content in reference to "female residents in bikinis with hands pointing to their breasts".

Same descriptions of categories potentially unprofessional and clearly unprofessional were used in Langenfeld et al's later publication [18], where they describe examples of questionable attire as men and women in revealing swimwear, and a group of men dressed in short skirts after losing a golf bet.

Koo's et al rubric for assessment of unprofessional content on FB has 35 categories of unprofessional content and potentially objectionable content [20]. In the potentially objectionable category, an image can be coded (among other sub-categories) as *inappropriate* or *offensive attire* or as *appearing in sexually suggestive attire or circumstances*, with no specific explanations (or differentiations) for either of the sub-categories. Koo et al designed [20] the rubric for assessment of unprofessional content on FB based on online professionalism guidelines by the American Urological Association (AUA) [52], the AMA [6] and the ACGME [51].

Hardouin et al's paper [22], also used Koo's et al research [20,21], and their rubric for assessment of unprofessional content on FB, as the basis for assessment of unprofessional content on FB, to allow them a direct comparison as possible between results.

Potentially unprofessional content was therefore similarly described in their al paper [22] as: holding/consuming alcohol, inappropriate attire, censored profanity, controversial political or religious comments, and controversial social topics. Their description of *inappropriate attire* included pictures in underwear, provocative Halloween costumes, and provocative posing in bikinis/swimwear. Besides the controversial word *bikin*i, there is the second semantical part of the controversy in the syntax used in that paper, *provocative* posing in bikinis/swimwear. That second part is the adjective *provocative* in the context of wearing bikinis/swimwear, which was overlooked and created the #medbikini movement. Hardouin et al [22] never mentioned that every picture in a bikini is potentially unprofessional. This is one example where the problem of the lack of precise definition is clearly visible.

Several studies could be seen as predecessors of #medbikini. Due to mixed terminology and lack of precise descriptions, images of (not only) female bodies in swimwear/bikinis, in the context of appropriateness to be publicly available on DM, were coded varyingly. Either as a part of the *questionable* attire, within it as a revealing swimwear [17], or as *appearing in sexually suggestive attire or circumstances* [20] or as a part of *inappropriate attire*, within it as a provocative posing in bikinis/swimwear [22].

In a recently published paper (2021) by Pronk et al [49] that studied all levels of medical professionals (students, residents and specialists), authors found that all investigated groups perceived information or pictures to be unprofessional related to alcohol abuse, partying and sexually suggestive posts, creating a dissonance between the #medbiniki movements perception of professionalism and collected data. However, they argue that the opinion of some participants may have changed due to the debate initiated by the #medbikini movement, that took place after their data collection.

This is a Multimedia Appendix to a full manuscript published in the JMIR Med Educ. For full copyright and citation information see http://dx.doi.org/10.2196/35585.
